# Supplementary material for: Social status and the relationship between income rank and well-being in 109 nations
Source: Nat Commun. 2026 Feb 20;17:2962. doi: 10.1038/s41467-026-69729-x (PMC13035997; doi:10.1038/s41467-026-69729-x)
Supplement: Supplementary file 1 — Supplementary Information [file 41467_2026_69729_MOESM1_ESM.pdf]

Supplementary Information

Social status and the relationship between income rank and well-being in 109 nations

Edika Quispe–Torreblanca, Jan-Emmanuel De Neve and Gordon D. A. Brown

Supplementary Note 1: Global Evidence of Income Rank Effects on Subjective Well-Being

Associations between income rank and subjective well-being have been found in single-nation studies in at least eight different countries. Income rank has been shown to be a better predictor of various measures of subjective well-being (SWB) in single-country datasets from Australia (e.g., 1, 2), China (e.g., 3), Denmark (e.g., 4), Germany (e.g., 5), India (e.g., 6), Japan (e.g., 7), Ecuador (e.g., 8), the UK (e.g., 9), and the USA (e.g., 10), and in one study of 24 countries [11].

Supplementary Note 2: Overview of Country-level Data Sources

The main data supporting the findings of this article are available from the Gallup Organization. Gallup data are accessible to researchers with a subscription or those who hold the status of a research advisor. (For more information, see https://www.gallup.com/analytics/213617/gallup-analytics.aspx.) We use the samples available across three pairs of consecutive survey rounds during the period 2013-2024: Rounds 8 (2013), 9 (2014–2015), 12 (2017–2018), 13 (2018–2019), 17 (2022–2023), and 18 (2023–2024). We report any data exclusions (if applicable). Additional data sources are described below.

- *General Country Characteristics.* We use log-transformed GDP per capita (PPP, current international dollars) and health expenditure per capita (PPP, current international dollars), unemployment rate (% of the total labor force), and urban population (% of the total population) as country-level indicators. These were extracted from the World Bank World Development Indicators Database based on the latest available estimates at the time of download (April 2025). As measures of income inequality, we extracted the Gini index and the level of absolute redistribution, both from the Standardized World Income Inequality Database [12]. Gini Index is a measure of inequality in disposable (post-tax, post-transfer) income. Absolute Redistribution is equivalent to the difference between the Gini index post-tax and the Gini index pre-tax.
- *Subjective Sentiment.* We use the following GWP indices as a proxy of societal subjective sentiment in a diverse set of political, social, and economic topics: community basics index, corruption index, national institutions index, civic engagement index, law and order index, social life index, and migrant acceptance index. When data

on Migrant Acceptance were not available for Round 18 (2023–2024), we used values from the preceding round (Round 17). Country-level variables were obtained by averaging individual responses for each country × year.

- *Individualism.* We constructed two measures of individualism from the World Values Survey (WVS) [13]: an autonomy index and an indicator of the importance of friends relative to the family (used also by 14). In the WVS, respondents received a list of qualities that children can be encouraged to learn at home. The autonomy index was calculated by summing the importance ratings of independence and determination, and subtracting the ratings for religious faith and obedience. The WVS respondents were also asked about the importance of their friends and family. We construct our second indicator by subtracting the score for the family from that for friends. Responses were reverse coded from the original scale (1 = very important, 4 = not very important). Both indicators were constructed using data from the most recent available wave of the WVS (2017–2022).
- *Materialism.* We constructed three measures of materialism from the WVS. The first is based on two Schwartz items that capture personal values. Respondents were asked to rate how similar they are to a person described as (1) "It is important to this person to be rich; to have a lot of money and expensive things," and (2) "Being very successful is important to this person; to have people recognize one's achievements." We averaged responses to these two items to construct a composite measure of the importance placed on wealth and success. Data for this indicator come from the most recent WVS wave in which both items were available (2010–2014).

  The second focuses on the trade-off between work and leisure. Respondents were asked to rate how important each of the following aspects is in their life: "Work" and "Leisure time." We constructed an index by subtracting the leisure score from the work score, so that higher values reflect a stronger orientation toward work.

  We included as a third measure of materialism the WVS 12-item Inglehart post-materialism score, which we labeled the Materialist Index (reverse coded so that higher scores indicated greater materialist values, such as the desire for economic growth and physical insecurity, relative to postmaterialist values, such as preferences for self-expression and for participation in political processes and decision-making). Data for these two measures come from the most recent WVS wave (2017–2022).

  Measures of individualism and materialism were available for 50 countries, except for the first materialism indicator (importance placed on wealth and success), which was available for only 43 countries due to data limitations in the 2010–2014 wave. All WVS country-level variables were obtained by averaging individual responses for each country.
- *Social Preferences.* We obtained country-level measures of social preferences from the Global Preferences Survey [15]. The GPS is a nationally representative dataset that includes measures of time preference, risk preference, and trust, as well as measures for three distinct dimensions of social preferences: altruism, positive reciprocity, and negative reciprocity. The GPS data were collected within the framework of the 2012 GWP (Round 7). Social preferences are estimated from standard incentivized economic choice experiments and non-incentivized surveys. The analysis

examining associations with social preferences included data from 65 countries that were successfully matched.

- *Economic Competitiveness*. We extracted market competition indicators across six pillars from the 2019 Global Competitiveness Report [16]: institutions (capturing property rights, ethics and corruption, public sector performance, and corporate governance and accountability), higher education (capturing enrollment rates, quality of the education system, and the extent of training of the workforce), goods market (capturing domestic competition, openness to foreign firms, and the degree of customer orientation), labor market (capturing hiring and firing practices, and the country's capacity to attract and retain talent), business sophistication (capturing the quantity and quality of local suppliers, business networks, and the individual firms' operation and strategies), and innovation capability (capturing the capacity for innovation, quality of scientific research institutions, and investment in R&D). We included data from 102 successfully matched countries.
- *Economic Openness*. We extracted measures of economic openness from the 2023 Legatum Prosperity Index Report covering four pillars [17]: market access and infrastructure (capturing the quality of communications, transport and energy, import tariff barriers, and market distortions), investment environment (capturing property rights, investor protections, and the financing ecosystem), enterprise conditions (capturing domestic market contestability, and the environment for business creation) and governance (capturing government integrity and effectiveness, political accountability, and the rule of law). For each country, we averaged scores from 2013 and 2023 to capture long-term structural conditions. Data were included for 107 matched countries.

To standardize the key metrics, we z-scored all country-level variables in the sample (except for the general country characteristics). Because most country-level variables were only available for certain years, when we study the moderating role of these indicators on the income rank effect, we base our analysis on GWP Round 18, 2023-2024, for which we could use the full set of variables—limiting the risk of potential reverse causality in our econometric specifications if we were to condition life evaluation of earlier survey rounds on controls variables collected during posterior years. Supplementary Table 1 presents a correlation matrix for the country-level variables used in the paper.

Supplementary Note 3: Individual-level Variables and Data Sources

To complement our country-level indicators, we also recover individual-level scores from the GWP covering analogous dimensions of analyses. We used individual-level data from the GWP as follows:

- *Demographics*. We obtained from the GWP the following demographic variables: age, gender, education, labor status, marital status, urban or rural condition, and self-reported health status.

- *Subjective Sentiment.* Likewise, we use the following GWP indices as a proxy of societal subjective sentiment in a diverse set of political, social, and economic topics: community basics index, corruption index, national institutions index, civic engagement index, law and order index, and social life index.
- *Social Preferences.* We use individual measures of social preferences from Round 7, 2012-2013, of the GWP, including time preference, risk preference, trust, altruism, positive reciprocity, and negative reciprocity.

Supplementary Note 4: Robustness Analyses

First, in our main analysis (Table 1), we address missing covariate data by imputing zeros for missing values and including a corresponding indicator variable. This approach preserves country coverage but may introduce bias if data are not missing at random. To address this concern, Supplementary Table 8 reports results from a complete-case analysis, restricting the sample to countries with no missing values in either country- or individual-level covariates. The results are very similar to those of the main specification.

Second, in Supplementary Table 7, we replicate the main results with country fixed effects included. The coefficient on income rank remains stable and statistically significant, while the coefficient on absolute income becomes negative and non-significant. This likely reflects the fact that, with fixed effects included, absolute income is effectively centered within countries. As a result, its coefficient no longer captures cross-country variation in absolute income, but rather income differences relative to each country's mean. This makes it conceptually similar to income rank and introduces substantial collinearity between the two measures ($r = 0.97$), inflating standard errors and limiting interpretability.

Third, we test the sensitivity of our results to the inclusion of covariates. In Supplementary Table 9, we estimate a series of models that gradually introduce individual- and country-level controls. Column 3 includes both log income and income rank without covariates. Column 4 adds individual-level controls (age, gender, and a fourth-degree polynomial of age interacted with gender). Column 5 introduces country-level variables weakly correlated with GDP per capita, while Column 6 adds those that are highly correlated (correlation > 0.7). We observe that when macro-level variables are excluded, the coefficient on absolute income is statistically significant. However, this likely reflects the fact that, in the absence of contextual controls, absolute income partially captures country-level differences in infrastructure, services, and general development—factors that extend beyond individual purchasing power. In other words, without appropriate controls, the absolute income coefficient conflates personal material resources with national living standards, making interpretation ambiguous. As shown in Supplementary Table 9, once country-level controls are included, income rank consistently emerges as a stronger predictor of SWB than absolute income. This pattern supports our interpretation that relative social position plays a more prominent role in shaping well-being once basic needs and broader contextual conditions are accounted for.

Fourth, we examine the sensitivity of our results to the income measure used. Our main analyses use PPP-adjusted household income, unadjusted for household size. In

Supplementary Table 10, we replicate the models in Supplementary Table 9 using per capita household income, calculated by Gallup as total household income divided by household size. When income rank is based on equivalized income, it no longer significantly predicts SWB. This suggests that individuals evaluate their social status using unadjusted (raw) income rather than income adjusted for household composition. While equivalized income better reflects material well-being, social comparisons appear to be based on visible, culturally salient indicators, such as raw income, which aligns with how income is commonly discussed, displayed, and institutionalized (e.g., tax brackets, salaries, job titles). Estimating equivalized income, in contrast, requires knowledge of household structure, which is not socially prominent.

Finally, we examine whether associations differ by national income level by splitting the sample at the median GDP per capita and re-estimating the models from Table 1 (Supplementary Table 11). Columns 1–3 report results for lower-income countries, and Columns 4–6 for higher-income ones. Absolute income has a larger coefficient in lower-income countries, where it is more directly tied to meeting basic needs. In contrast, income rank shows a stronger association with well-being in higher-income countries, where income more often signals social status and discretionary spending. Crucially, income rank remains statistically significant in both groups, whereas the association between absolute income and SWB becomes non-significant once controls are included, particularly in wealthier contexts. These results are consistent with the idea that social status measured by relative income plays a more prominent role in shaping life evaluation as material conditions improve.

Supplementary Note 5: Income Rank, Inequality and Subjective Well-being

In Analysis 3, we noted that the associations of income rank and SWB do not appear to be determined by inequality. Our estimates show a non-significant positive income rank-inequality interaction. This result is consistent with a recent meta-analysis of 357 studies investigating moderators of the association between subjective well-being and subjective socioeconomic status (the latter referring to rank-based judgements relative to any comparison target, e.g., the whole country, the local community, the group of co-workers, etc.) [18]. The meta-analysis reports null interaction effects regardless of how income inequality was assessed, except when GINI was used as an inequality measure. In this case, the social position effect decreased as GINI increased.

Although our results are in agreement with those obtained by [18], our results differ from an earlier study documenting a positive interaction between income rank and inequality on subjective well-being in a subset of 24 countries of the GWP, covering the period from 2009 to 2015 [11]. This inconsistency may be due to the different sample sizes and time frame that we examine. For completeness, we reproduce Macchia et al.'s findings for their smaller subset of countries. We study fifteen years, from 2009 to 2024. Supplementary Figure 17 presents unconditional plots of the income rank-inequality interaction; we find a positive interaction in only 3 out of 15 years (2010, 2013, and 2015). When we extend the sample to cover 113 countries, in Supplementary Figure 18, we find a positive interaction in only three years (2010, 2015, and 2022). Because for this exercise we only study unconditional patterns, we are able to analyse

a larger set of countries than the set we study in our main analyses, as we are not restricted by the availability of control variables for the analysis. In Supplementary Figure 18, the interaction coefficient changes sign erratically over time. This pattern of results suggests that inequality plays, if anything, a minor moderating role in income rank's association with SWB.

Supplementary Note 6: Extended Regression Results for Main Tables

Supplementary Tables 23–26 report the full regression outputs underlying Tables 1 and 2 in the main manuscript. These extended tables include all coefficients and p-values for the complete set of control variables. Because the full models are extensive, the main manuscript presents only the key coefficients for readability, while the complete results are provided here. Supplementary Table 23 corresponds to Table 1, and Supplementary Tables 24–26 correspond to Table 2, reporting the associations between income or income rank and the alternative measures of well-being.

Supplementary Table 1: Correlation Matrix of Country Level Variables

	(1)	(2)	(3)	(4)	(5)	(6)	(7)	(8)	(9)	(10)	(11)	(12)	(13)	(14)	(15)	(16)	(17)	(18)	(19)
1: Life Evaluation (0-10)	1																		
2: Life Evaluation in 5 Years (0-10)	0.63	1																	
3: Positive Affect (0-1)	0.47	0.6	1																
4: Negative Affect (0-1)	-0.47	-0.28	-0.3	1															
5: Ln GDP p.c. ($1000)	**0.76**	0.21	0.16	-0.41	1														
6: Ln Health Expenditure p.c. ($1000)	**0.74**	0.14	0.15	-0.39	**0.96**	1													
7: Unemployment (%)	-0.15	-0.12	-0.15	0.1	0.04	0.12	1												
8: Urban Population (%)	0.65	0.36	0.2	-0.14	**0.74**	**0.74**	0.24	1											
9: Gini Index (0-100)	-0.48	0.1	0.18	0.4	-0.58	-0.56	0.04	-0.29	1										
10: Absolute Redistribution	0.53	-0.18	-0.25	-0.41	**0.79**	**0.8**	0.01	0.28	**-0.77**	1									
11: Autonomy Index	0.58	0.14	0.18	-0.63	0.63	0.65	-0.4	0.36	-0.56	0.62	1								
12: Importance of Friends vs Family	0.35	-0.14	-0.16	-0.31	0.47	0.48	0.11	0.28	-0.45	**0.72**	0.5	1							
13: Materialist Index	-0.6	-0.22	-0.36	0.17	-0.46	-0.45	0.3	-0.35	0.1	-0.39	-0.44	-0.33	1						
14: Importance to be Rich/Successful	-0.54	-0.22	-0.31	0.29	-0.54	-0.59	0.45	-0.41	0.17	-0.43	-0.54	-0.14	0.33	1					
15: Importance of Work vs Leisure Time	-0.64	-0.06	-0.05	0.37	**-0.78**	**-0.81**	0.09	-0.63	0.45	-0.68	-0.65	-0.63	0.46	0.48	1				
16: Institutions	0.6	0.09	0.09	-0.37	**0.78**	**0.79**	0.03	0.57	-0.54	**0.76**	0.66	0.56	-0.48	-0.57	-0.66	1			
17: Higher Education	**0.72**	0.14	0.07	-0.4	**0.9**	**0.92**	0.08	0.67	-0.57	**0.77**	0.7	0.47	-0.4	-0.56	**-0.72**	**0.85**	1		
18: Goods Market	0.65	0.24	0.19	-0.35	**0.72**	**0.74**	-0.02	0.6	-0.4	0.58	0.6	0.4	-0.44	-0.54	-0.62	**0.85**	**0.77**	1	
19: Labor Market	0.55	0.11	0.1	-0.42	0.67	0.69	-0.07	0.44	-0.53	0.67	**0.72**	0.49	-0.49	-0.55	-0.64	**0.82**	**0.78**	**0.79**	1
20: Business Sophistication	0.58	0.1	0.11	-0.36	**0.71**	**0.71**	-0.04	0.49	-0.48	**0.72**	0.66	0.55	-0.52	-0.52	-0.59	**0.86**	**0.79**	**0.8**	**0.82**
21: Innovation	0.62	0.06	0.08	-0.26	**0.75**	**0.78**	-0.09	0.57	-0.53	**0.73**	0.7	0.54	-0.5	-0.55	-0.65	**0.87**	**0.78**	**0.74**	**0.72**
22: Market Access Infrastructure	**0.73**	0.15	0.2	-0.36	**0.92**	**0.94**	0.06	0.69	-0.55	**0.8**	0.63	0.44	-0.49	-0.6	**-0.72**	**0.86**	**0.9**	**0.82**	**0.72**
23: Investment Environment	0.66	0.11	0.2	-0.35	**0.86**	**0.88**	0.08	0.66	-0.5	**0.78**	0.61	0.52	-0.53	-0.6	**-0.73**	**0.92**	**0.87**	**0.81**	**0.77**
24: Enterprise Conditions	0.55	0.07	0.15	-0.28	**0.74**	**0.76**	-0.01	0.54	-0.43	**0.71**	0.61	0.52	-0.49	-0.55	-0.64	**0.92**	**0.77**	**0.84**	**0.79**
25: Governance	0.62	0.13	0.2	-0.36	**0.77**	**0.8**	0.03	0.6	-0.51	**0.74**	0.68	0.56	-0.57	-0.66	**-0.72**	**0.92**	**0.8**	**0.77**	**0.76**
26: Patience	0.44	0.14	0.14	-0.25	0.55	0.6	-0.16	0.36	-0.45	0.46	0.63	0.42	-0.57	-0.54	-0.55	0.7	0.63	0.61	0.67
27: Risk Taking	-0.13	0.02	0.06	0	-0.09	-0.07	0.34	-0.01	0.23	-0.15	0.12	0.14	-0.32	0.21	-0.08	0.08	0	0.07	0.14
28: Positive Reciprocity	-0.11	-0.12	-0.29	0.22	0.01	0.07	-0.06	-0.05	-0.08	0.14	-0.1	-0.03	0.18	-0.04	0.15	0.01	0.1	-0.03	-0.02
29: Negative Reciprocity	0.11	-0.2	-0.22	-0.1	0.31	0.26	-0.07	0.12	-0.28	0.18	0.32	0.26	-0.06	-0.06	-0.16	0.18	0.23	0.14	0.04
30: Altruism	-0.24	-0.08	-0.19	0.31	-0.11	-0.09	0.05	-0.09	0.2	-0.19	-0.17	-0.21	0.1	0	0.31	-0.04	-0.06	-0.07	-0.13
31: Trust	0.2	0.01	-0.04	-0.02	0.32	0.3	0	0.24	-0.24	0.23	-0.21	0.05	0.09	0.11	0.12	0.38	0.39	0.36	0.25
32: Community Basics Index	0.45	0.38	0.45	-0.32	0.3	0.28	-0.27	0.08	-0.16	0.24	0.26	-0.06	-0.26	-0.25	0.1	0.4	0.33	0.42	0.37
33: Corruption Index	-0.42	-0.15	-0.08	0.28	-0.41	-0.42	0.15	-0.37	0.42	-0.53	-0.44	-0.42	0.34	0.49	0.47	-0.66	-0.5	-0.6	-0.61
34: National Institutions Index	0.06	0.14	0.11	-0.09	0.01	-0.05	-0.15	-0.04	-0.16	0.48	0.2	0.29	-0.16	-0.09	0.02	0.35	0.07	0.29	0.23
35: Civic Engagement Index	0.23	0.41	0.39	0.01	0.08	0.05	-0.21	0.06	0.08	0.15	0.13	0.15	-0.28	-0.19	-0.06	0.2	0.13	0.18	0.18
36: Law and Order Index	0.44	0.05	-0.03	-0.38	0.53	0.53	-0.09	0.24	-0.58	0.62	0.35	0.43	0	-0.05	-0.13	0.58	0.57	0.56	0.5
37: Social Life Index	**0.77**	0.54	0.55	-0.51	0.6	0.6	-0.1	0.39	-0.34	0.35	0.43	0.23	-0.36	-0.4	-0.39	0.42	0.59	0.44	0.48
38: Migrant Acceptance Index	0.21	0.36	0.36	-0.04	0.03	0.03	-0.31	0.14	-0.03	0.17	0.41	0.14	-0.28	-0.68	-0.16	0.24	0.06	0.25	0.16

Note: Correlation matrix for the country level variables used in the main analyses of the paper. For comparison, the table starts by presenting country-level measures of life evaluation and hedonic well-being using Round 18 (2023–2024) of the Gallup World Poll (variables 1 to 4). The remaining country-level variables correspond to the year preceding the survey administration, or the most recent available year within the past five. Variables 11 to 38 are standardized across countries. Correlation scores above .7 are bolded. Sources are described above.

Supplementary Table 1: Correlation Matrix of Country Level Variables (Continuation)

	(20)	(21)	(22)	(23)	(24)	(25)	(26)	(27)	(28)	(29)	(30)	(31)	(32)	(33)	(34)	(35)	(36)	(37)	(38)
20: Business Sophistication	1																		
21: Innovation	**0.84**	1																	
22: Market Access Infrastructure	**0.79**	**0.84**	1																
23: Investment Environment	**0.85**	**0.88**	**0.94**	1															
24: Enterprise Conditions	**0.85**	**0.89**	**0.86**	**0.93**	1														
25: Governance	**0.8**	**0.85**	**0.85**	**0.93**	**0.92**	1													
26: Patience	0.68	**0.78**	0.61	**0.71**	**0.72**	**0.74**	1												
27: Risk Taking	0	-0.03	-0.13	0.04	0.04	0.07	0.3	1											
28: Positive Reciprocity	0.04	-0.03	0.01	-0.05	-0.04	-0.07	-0.07	-0.29	1										
29: Negative Reciprocity	0.15	0.28	0.25	0.2	0.16	0.13	0.29	0.21	-0.14	1									
30: Altruism	-0.05	-0.06	-0.1	-0.13	-0.05	-0.11	-0.05	-0.07	**0.71**	-0.13	1								
31: Trust	0.3	0.27	0.29	0.22	0.18	0.13	0.17	-0.03	0.29	0.17	0.21	1							
32: Community Basics Index	0.36	0.36	0.38	0.34	0.37	0.31	0.39	-0.04	-0.11	0.06	0	0.23	1						
33: Corruption Index	-0.54	-0.59	-0.47	-0.52	-0.59	-0.61	-0.53	0.08	0.05	0	0.07	-0.36	-0.48	1					
34: National Institutions Index	0.32	0.29	0.1	0.21	0.38	0.31	0.28	-0.03	-0.14	-0.07	-0.01	0.21	0.52	-0.56	1				
35: Civic Engagement Index	0.22	0.2	0.08	0.12	0.18	0.15	0.24	0.11	0.04	-0.07	0.09	0.19	0.3	-0.05	0.22	1			
36: Law and Order Index	0.54	0.49	0.57	0.51	0.52	0.46	0.3	-0.34	0.16	0.28	0.04	0.57	0.56	-0.52	0.47	-0.01	1		
37: Social Life Index	0.44	0.4	0.56	0.51	0.41	0.44	0.33	-0.13	-0.08	0.02	-0.23	0.19	0.52	-0.25	0	0.32	0.39	1	
38: Migrant Acceptance Index	0.15	0.26	0.08	0.12	0.22	0.27	0.42	-0.03	-0.02	-0.17	0.22	-0.01	0.28	-0.34	0.42	0.37	0.04	0.07	1

Note: Correlation matrix for the country level variables used in the main analyses of the paper. For comparison, the table starts by presenting country-level measures of life evaluation and hedonic well-being using Round 18 (2023–2024) of the Gallup World Poll (variables 1 to 4). The remaining country-level variables correspond to the year preceding the survey administration, or the most recent available year within the past five. Variables 11 to 38 are standardized across countries. Correlation scores above .7 are bolded. Sources are described above.

Supplementary Table 2: Descriptive Statistics

	Quartile 1, GDP		Quartile 2, GDP		Quartile 3, GDP		Quartile 4, GDP	
	Mean	SD	Mean	SD	Mean	SD	Mean	SD
Life Evaluation (0 to 10)	4.63	2.72	5.45	2.57	6.12	2.13	6.83	1.85
Income ($1000)	4.64	4.52	9.12	7.54	20.45	15.75	45.88	32.27
Age (years)	35.71	15.36	42.57	17.57	48.09	17.95	49.82	18.11
Gender (Female)	0.54		0.59		0.57		0.49	
Educational Level:								
Elementary Education	0.50		0.29		0.18		0.06	
Secondary Education	0.43		0.58		0.58		0.58	
College Degree	0.07		0.13		0.24		0.36	
Employment Status:								
Employed Full Time	0.17		0.22		0.38		0.41	
Employed Full Time (for self)	0.23		0.15		0.09		0.06	
Employed Part Time (Do Not Want Full Time)	0.08		0.07		0.07		0.07	
Employed Part Time (Want Full Time)	0.12		0.09		0.06		0.05	
Out of Workforce	0.32		0.39		0.36		0.38	
Unemployed	0.08		0.08		0.04		0.04	
Marital Status:								
Single	0.29		0.32		0.25		0.27	
Married	0.53		0.44		0.44		0.50	
Domestic Partner	0.06		0.08		0.09		0.07	
Divorced	0.03		0.03		0.07		0.07	
Separated	0.03		0.03		0.03		0.02	
Widowed	0.06		0.09		0.11		0.07	
Urban/Rural:								
Large City	0.19		0.32		0.38		0.36	
Suburb of a Large City	0.09		0.12		0.09		0.17	
Small Town or Village	0.35		0.31		0.36		0.32	
Rural Area	0.36		0.25		0.17		0.14	
Observations	24995		23995		24429		23920	

Note: Descriptive statistics of the main sample, Round 18 (2023–2024) of the Gallup World Poll. Observations are split by quartile of GDP per-capita across columns 1 to 4.

Supplementary Table 3: Income Effects on Life Evaluation Across Survey Rounds, Pooling Countries

	Round 8: 2013				
	(1)	(2)	(3)	(4)	(5)
Log Income $1000	0.847***	0.442***			0.140
	(0.756,0.937)	(0.336,0.548)			(-0.096,0.376)
Income Rank Index (0-1)			1.550***	1.227***	0.936***
			(1.446,1.655)	(1.125,1.328)	(0.445,1.427)
Constant	3.522***	6.322***	4.675***	6.948***	6.662***
	(3.257,3.786)	(4.655,7.989)	(4.442,4.908)	(5.414,8.482)	(5.034,8.290)
Individual Level Controls	NO	YES	NO	YES	YES
Country Level Controls	NO	YES	NO	YES	YES
Observations	94,554	94,554	94,554	94,554	94,554
R^2	0.170	0.241	0.038	0.244	0.245

	Round 12: 2017-2018				
	(1)	(2)	(3)	(4)	(5)
Log Income $1000	0.838***	0.463***			0.302*
	(0.744,0.933)	(0.375,0.551)			(0.010,0.595)
Income Rank Index (0-1)			1.442***	1.117***	0.479
			(1.335,1.550)	(1.018,1.215)	(-0.164,1.122)
Constant	3.613***	7.025***	4.893***	7.804***	7.207***
	(3.313,3.914)	(5.404,8.646)	(4.658,5.129)	(6.231,9.377)	(5.563,8.850)
Individual Level Controls	NO	YES	NO	YES	YES
Country Level Controls	NO	YES	NO	YES	YES
Observations	99,333	99,333	99,333	99,333	99,333
R^2	0.154	0.211	0.029	0.209	0.211

	Round 17: 2022-2023				
	(1)	(2)	(3)	(4)	(5)
Log Income $1000	0.687***	0.330***			0.120
	(0.587,0.787)	(0.226,0.435)			(-0.134,0.375)
Income Rank Index (0-1)			1.349***	1.034***	0.772*
			(1.216,1.482)	(0.907,1.160)	(0.176,1.367)
Constant	4.064***	6.689***	5.020***	6.981***	6.771***
	(3.730,4.399)	(4.878,8.501)	(4.769,5.272)	(5.269,8.693)	(4.939,8.603)
Individual Level Controls	NO	YES	NO	YES	YES
Country Level Controls	NO	YES	NO	YES	YES
Observations	96,429	96,429	96,429	96,429	96,429
R^2	0.127	0.185	0.023	0.188	0.188

Note: OLS models for the effect of income and income rank. Columns 2, 4, and 5 control for age, gender (a four-degree polynomial of age and its interaction with gender), employment status, education, marital status, self-reported health, and urban/rural areas. Outliers below the 5 percentile and above the 95 percentile of incomes are excluded. Inference is based on two-sided t-tests with standard errors clustered at the country level; 95% confidence intervals are shown in parentheses. No multiple-comparison adjustments applied. $^{*}p<0.05$; $^{**}p<0.01$; $^{***}p<0.001$.

Supplementary Table 4: Income Effects on Life Evaluation in Five Years Across Survey Rounds, Pooling Countries

	Round 8: 2013				
	(1)	(2)	(3)	(4)	(5)
Log Income $1000	0.441***	0.426***			0.120
	(0.349,0.534)	(0.314,0.537)			(-0.149,0.389)
Income Rank Index (0-1)			1.653***	1.185***	0.936**
			(1.519,1.786)	(1.068,1.302)	(0.362,1.510)
Constant	5.712***	4.428***	5.897***	5.116***	4.849***
	(5.424,6.001)	(2.658,6.197)	(5.672,6.123)	(3.503,6.730)	(3.190,6.507)
Individual Level Controls	NO	YES	NO	YES	YES
Country Level Controls	NO	YES	NO	YES	YES
Observations	87,209	87,209	87,209	87,209	87,209
R^2	0.044	0.165	0.040	0.169	0.169

	Round 12: 2017-2018				
	(1)	(2)	(3)	(4)	(5)
Log Income $1000	0.464***	0.452***			0.292
	(0.345,0.584)	(0.357,0.546)			(-0.022,0.605)
Income Rank Index (0-1)			1.592***	1.080***	0.471
			(1.452,1.731)	(0.959,1.202)	(-0.221,1.163)
Constant	5.720***	5.141***	6.051***	5.893***	5.319***
	(5.313,6.127)	(3.227,7.054)	(5.822,6.281)	(4.011,7.775)	(3.325,7.314)
Individual Level Controls	NO	YES	NO	YES	YES
Country Level Controls	NO	YES	NO	YES	YES
Observations	91,640	91,640	91,640	91,640	91,640
R^2	0.046	0.164	0.035	0.163	0.165

	Round 17: 2022-2023				
	(1)	(2)	(3)	(4)	(5)
Log Income $1000	0.282***	0.336***			0.181
	(0.174,0.390)	(0.214,0.458)			(-0.140,0.501)
Income Rank Index (0-1)			1.537***	0.965***	0.573
			(1.378,1.696)	(0.821,1.110)	(-0.200,1.346)
Constant	6.364***	5.779***	6.274***	6.163***	5.843***
	(6.006,6.722)	(3.564,7.994)	(6.062,6.486)	(4.132,8.194)	(3.544,8.141)
Individual Level Controls	NO	YES	NO	YES	YES
Country Level Controls	NO	YES	NO	YES	YES
Observations	89,868	89,868	89,868	89,868	89,868
R^2	0.022	0.138	0.030	0.138	0.139

Note: OLS models for the effect of income and income rank. Columns 2, 4, and 5 control for age, gender (a four-degree polynomial of age and its interaction with gender), employment status, education, marital status, self-reported health, and urban/rural areas. Outliers below the 5 percentile and above the 95 percentile of incomes are excluded. Inference is based on two-sided t-tests with standard errors clustered at the country level; 95% confidence intervals are shown in parentheses. No multiple-comparison adjustments applied. *p<0.05; **p<0.01; ***p<0.001.

Supplementary Table 5: Income Effects on Positive Affect Across Survey Rounds, Pooling Countries

	Round 8: 2013				
	(1)	(2)	(3)	(4)	(5)
Log Income $1000	0.036***	0.036***			-0.015
	(0.025,0.047)	(0.026,0.047)			(-0.045,0.014)
Income Rank Index (0-1)			0.166***	0.127***	0.159***
			(0.147,0.186)	(0.112,0.142)	(0.096,0.222)
Constant	0.637***	0.697***	0.635***	0.726***	0.757***
	(0.599,0.674)	(0.527,0.867)	(0.609,0.660)	(0.569,0.882)	(0.582,0.932)
Individual Level Controls	NO	YES	NO	YES	YES
Country Level Controls	NO	YES	NO	YES	YES
Observations	90,456	90,456	90,456	90,456	90,456
R^2	0.011	0.076	0.016	0.080	0.080

	Round 12: 2017-2018				
	(1)	(2)	(3)	(4)	(5)
Log Income $1000	0.038***	0.028***			-0.037*
	(0.027,0.049)	(0.018,0.038)			(-0.072,-0.002)
Income Rank Index (0-1)			0.155***	0.117***	0.195***
			(0.136,0.174)	(0.100,0.133)	(0.114,0.276)
Constant	0.615***	0.821***	0.629***	0.817***	0.886***
	(0.578,0.653)	(0.614,1.028)	(0.603,0.655)	(0.614,1.020)	(0.670,1.102)
Individual Level Controls	NO	YES	NO	YES	YES
Country Level Controls	NO	YES	NO	YES	YES
Observations	97,481	97,481	97,481	97,481	97,481
R^2	0.013	0.072	0.013	0.076	0.078

	Round 17: 2022-2023				
	(1)	(2)	(3)	(4)	(5)
Log Income $1000	0.024***	0.021*			-0.009
	(0.012,0.035)	(0.004,0.037)			(-0.052,0.033)
Income Rank Index (0-1)			0.136***	0.091***	0.111*
			(0.118,0.154)	(0.073,0.109)	(0.009,0.212)
Constant	0.657***	0.790***	0.645***	0.786***	0.802***
	(0.617,0.696)	(0.547,1.033)	(0.619,0.671)	(0.574,0.997)	(0.551,1.053)
Individual Level Controls	NO	YES	NO	YES	YES
Country Level Controls	NO	YES	NO	YES	YES
Observations	95,436	95,436	95,436	95,436	95,436
R^2	0.007	0.058	0.010	0.060	0.060

Note: OLS models for the effect of income and income rank. Columns 2, 4, and 5 control for age, gender (a four-degree polynomial of age and its interaction with gender), employment status, education, marital status, self-reported health, and urban/rural areas. Outliers below the 5 percentile and above the 95 percentile of incomes are excluded. Inference is based on two-sided t-tests with standard errors clustered at the country level; 95% confidence intervals are shown in parentheses. No multiple-comparison adjustments applied. *p<0.05; **p<0.01; ***p<0.001.

Supplementary Table 6: Income Effects on Negative Affect Across Survey Rounds, Pooling Countries

			Round 8: 2013		
	(1)	(2)	(3)	(4)	(5)
Log Income $1000	-0.015**	-0.029***			-0.000
	(-0.025,-0.005)	(-0.040,-0.018)			(-0.033,0.032)
Income Rank Index (0-1)			-0.108***	-0.090***	-0.089*
			(-0.123,-0.093)	(-0.106,-0.074)	(-0.163,-0.015)
Constant	0.315***	0.248*	0.335***	0.216*	0.217*
	(0.286,0.343)	(0.040,0.456)	(0.315,0.354)	(0.022,0.410)	(0.003,0.431)
Individual Level Controls	NO	YES	NO	YES	YES
Country Level Controls	NO	YES	NO	YES	YES
Observations	93,362	93,362	93,362	93,362	93,362
R^2	0.003	0.059	0.010	0.061	0.061
			Round 12: 2017-2018		
	(1)	(2)	(3)	(4)	(5)
Log Income $1000	-0.047***	-0.030***			0.005
	(-0.054,-0.039)	(-0.039,-0.022)			(-0.021,0.031)
Income Rank Index (0-1)			-0.124***	-0.096***	-0.106***
			(-0.140,-0.109)	(-0.109,-0.082)	(-0.164,-0.049)
Constant	0.413***	0.080	0.364***	0.054	0.045
	(0.389,0.438)	(-0.094,0.255)	(0.345,0.384)	(-0.109,0.218)	(-0.139,0.228)
Individual Level Controls	NO	YES	NO	YES	YES
Country Level Controls	NO	YES	NO	YES	YES
Observations	98,186	98,186	98,186	98,186	98,186
R^2	0.027	0.088	0.012	0.091	0.091
			Round 17: 2022-2023		
	(1)	(2)	(3)	(4)	(5)
Log Income $1000	-0.045***	-0.028***			-0.015
	(-0.054,-0.036)	(-0.039,-0.016)			(-0.044,0.014)
Income Rank Index (0-1)			-0.115***	-0.078***	-0.046
			(-0.131,-0.098)	(-0.093,-0.064)	(-0.117,0.024)
Constant	0.417***	0.028	0.368***	-0.001	0.024
	(0.390,0.445)	(-0.180,0.236)	(0.346,0.390)	(-0.206,0.205)	(-0.189,0.238)
Individual Level Controls	NO	YES	NO	YES	YES
Country Level Controls	NO	YES	NO	YES	YES
Observations	95,118	95,118	95,118	95,118	95,118
R^2	0.031	0.089	0.010	0.090	0.090

Note: OLS models for the effect of income and income rank. Columns 2, 4, and 5 control for age, gender (a four-degree polynomial of age and its interaction with gender), employment status, education, marital status, self-reported health, and urban/rural areas. Outliers below the 5 percentile and above the 95 percentile of incomes are excluded. Inference is based on two-sided *t*-tests with standard errors clustered at the country level; 95% confidence intervals are shown in parentheses. No multiple-comparison adjustments applied. *p<0.05; **p<0.01; ***p<0.001.

Supplementary Table 7: Income Effects on Life Evaluation, Country Fixed Effects Specifications

	Life Evaluation				
	(1)	(2)	(3)	(4)	(5)
Log Income $1000	0.514***	0.394***			-0.057
	(0.428,0.600)	(0.317,0.471)			(-0.195,0.082)
Income Rank Index (0-1)			1.385***	1.100***	1.229***
			(1.239,1.531)	(0.964,1.235)	(0.917,1.540)
Female		0.183		0.177	0.175
		(-0.784,1.149)		(-0.787,1.141)	(-0.789,1.140)
Education (Base: Elementary)					
College Degree		0.593***		0.550***	0.550***
		(0.508,0.678)		(0.471,0.629)	(0.471,0.629)
Secondary Education		0.274***		0.265***	0.267***
		(0.207,0.341)		(0.200,0.330)	(0.202,0.331)
Unreported		0.373**		0.368**	0.369**
		(0.115,0.630)		(0.112,0.624)	(0.113,0.624)
Employment Status (Base: Employed Full Time)					
Employed Full Time for Self		-0.069		-0.054	-0.053
		(-0.166,0.029)		(-0.150,0.042)	(-0.149,0.043)
Employed Part Time (do not want full time)		0.106**		0.119**	0.119**
		(0.034,0.178)		(0.049,0.190)	(0.049,0.190)
Employed Part Time (want full time)		-0.199***		-0.179***	-0.179***
		(-0.273,-0.124)		(-0.251,-0.108)	(-0.251,-0.108)
Unemployed		-0.494***		-0.468***	-0.468***
		(-0.594,-0.393)		(-0.565,-0.370)	(-0.565,-0.370)
Out of Workforce		-0.057		-0.034	-0.033
		(-0.118,0.004)		(-0.095,0.027)	(-0.095,0.028)
Health Problems (Base: Yes)					
No		0.565***		0.555***	0.556***
		(0.482,0.648)		(0.473,0.638)	(0.474,0.638)
Unreported		0.262		0.253	0.251
		(-0.086,0.611)		(-0.088,0.594)	(-0.089,0.591)
Marital Status (Base: Single)					
Married		0.150***		0.128***	0.127***
		(0.089,0.210)		(0.068,0.188)	(0.068,0.187)
Separated		-0.167**		-0.171**	-0.171**
		(-0.270,-0.063)		(-0.274,-0.068)	(-0.274,-0.068)
Divorced		-0.075		-0.063	-0.062
		(-0.151,0.001)		(-0.139,0.013)	(-0.139,0.014)
Widowed		-0.030		-0.024	-0.024
		(-0.117,0.056)		(-0.110,0.061)	(-0.110,0.061)
Domestic Partner		0.094*		0.079	0.078
		(0.015,0.174)		(-0.001,0.159)	(-0.001,0.158)
Unreported		0.289		0.308	0.309
		(-0.038,0.616)		(-0.016,0.631)	(-0.014,0.633)
Urban Area (Base: Rural Area)					
Small Town		0.006		0.004	0.005
		(-0.058,0.070)		(-0.058,0.067)	(-0.058,0.068)
Large City		0.038		0.028	0.029
		(-0.039,0.114)		(-0.048,0.104)	(-0.048,0.105)
Suburb of a Large City		0.012		0.002	0.003
		(-0.070,0.093)		(-0.079,0.084)	(-0.078,0.085)
Unreported		-0.364*		-0.362*	-0.361*
		(-0.688,-0.039)		(-0.690,-0.035)	(-0.689,-0.034)
Observations	97,339	97,339	97,339	97,339	97,339
R^2	0.210	0.236	0.214	0.238	0.238

Note: OLS models for the effect of income and income rank on life evaluation (for Survey Years 2023-2024). Columns 2, 4, and 5 incorporate individual level controls, including age, gender (a four-degree polynomial of age and its interaction with gender), and country level controls. Country level controls are for the year preceding the survey (or the most recent available year within the past five). Columns 2, 4 and 5 also include dummy variables for missing country level indicators. Outliers below the 5 percentile and above the 95 percentile of incomes are excluded. Inference is based on two-sided t-tests with standard errors clustered at the country level; 95% confidence intervals are shown in parentheses. No multiple-comparison adjustments applied. *p<0.05; **p<0.01; ***p<0.001.

Supplementary Table 8: Income Effects on Life Evaluation, Pooling Countries (Excludes Observations with Missing Data)

	Life Evaluation				
	(1)	(2)	(3)	(4)	(5)
Log Income $1000	0.584***	0.331***			-0.140
	(0.410,0.759)	(0.179,0.482)			(-0.388,0.109)
Income Rank Index (0-1)			1.447***	1.175***	1.454***
			(1.257,1.637)	(0.974,1.377)	(0.918,1.989)
Female		0.375		0.459	0.488
		(-1.009,1.759)		(-0.927,1.845)	(-0.919,1.895)
Education (Base: Elementary)					
College Degree		0.445***		0.385***	0.401***
		(0.313,0.576)		(0.255,0.515)	(0.269,0.534)
Secondary Education		0.232***		0.210***	0.220***
		(0.107,0.357)		(0.085,0.334)	(0.099,0.342)
Employment Status (Base: Employed Full Time)					
Employed Full Time for Self		-0.061		-0.047	-0.053
		(-0.220,0.099)		(-0.202,0.108)	(-0.208,0.102)
Employed Part Time (do not want full time)		0.159**		0.175***	0.166***
		(0.064,0.254)		(0.074,0.276)	(0.070,0.262)
Employed Part Time (want full time)		-0.100		-0.088	-0.107
		(-0.220,0.020)		(-0.207,0.030)	(-0.216,0.002)
Unemployed		-0.532***		-0.490***	-0.503***
		(-0.652,-0.412)		(-0.609,-0.371)	(-0.622,-0.384)
Out of Workforce		-0.094		-0.057	-0.062
		(-0.193,0.004)		(-0.153,0.039)	(-0.158,0.035)
Health Problems (Base: Yes)					
No		0.734***		0.720***	0.726***
		(0.647,0.821)		(0.637,0.803)	(0.643,0.810)
Marital Status (Base: Single)					
Married		0.091		0.077	0.092
		(-0.011,0.194)		(-0.023,0.178)	(-0.009,0.193)
Separated		-0.087		-0.112	-0.121
		(-0.245,0.071)		(-0.262,0.038)	(-0.269,0.028)
Divorced		-0.098		-0.054	-0.046
		(-0.223,0.027)		(-0.170,0.061)	(-0.162,0.071)
Widowed		-0.056		-0.039	-0.037
		(-0.195,0.084)		(-0.173,0.094)	(-0.169,0.096)
Domestic Partner		0.266***		0.219**	0.214**
		(0.122,0.410)		(0.088,0.350)	(0.086,0.343)
Urban Area (Base: Rural Area)					
Small Town		-0.147**		-0.142*	-0.135*
		(-0.259,-0.035)		(-0.256,-0.027)	(-0.249,-0.020)
Large City		-0.147*		-0.138*	-0.125
		(-0.286,-0.007)		(-0.277,-0.000)	(-0.261,0.012)
Suburb of a Large City		-0.047		-0.029	-0.010
		(-0.207,0.113)		(-0.187,0.129)	(-0.166,0.145)
Country Level Controls					
Ln Health Expenditure Per Capita $1000		0.151		0.322**	0.391**
		(-0.101,0.403)		(0.100,0.543)	(0.148,0.635)
Unemployment (% of total labor force)		-0.030		-0.032	-0.033
		(-0.089,0.030)		(-0.085,0.021)	(-0.085,0.019)
Urban Population (% of total population)		0.014*		0.015**	0.015**
		(0.002,0.025)		(0.004,0.026)	(0.004,0.026)
Gini Index (0-100)		0.017		0.002	-0.003
		(-0.013,0.047)		(-0.025,0.029)	(-0.030,0.024)
Absolute Redistribution		0.008		0.010	0.012
		(-0.031,0.047)		(-0.025,0.046)	(-0.023,0.046)
Constant	4.641***	5.277***	5.538***	5.716***	6.071***
	(4.041,5.240)	(3.466,7.088)	(5.229,5.847)	(3.809,7.623)	(4.277,7.865)
Observations	52,257	52,257	52,257	52,257	52,257
R^2	0.079	0.131	0.035	0.141	0.142

Note: OLS models for the effect of income and income rank on life evaluation (for Survey Years 2023-2024). Columns 2, 4, and 5 incorporate individual level controls, including age, gender (a four-degree polynomial of age and its interaction with gender), and country level controls. Country level controls are for the year preceding the survey (or the most recent available year within the past five). Outliers below the 5 percentile and above the 95 percentile of incomes are excluded. Inference is based on two-sided t-tests with standard errors clustered at the country level; 95% confidence intervals are shown in parentheses. No multiple-comparison adjustments applied. *p<0.05; **p<0.01; ***p<0.001.

Supplementary Table 9: Income Effects on Life Evaluation, Specifications with Incremental Controls

	Life Evaluation					
	(1)	(2)	(3)	(4)	(5)	(6)
Log Income $1000	0.663***<br>(0.570,0.756)		0.681***<br>(0.566,0.795)	0.566***<br>(0.461,0.672)	0.547***<br>(0.411,0.684)	0.068<br>(-0.129,0.266)
Income Rank Index (0-1)		1.385***<br>(1.239,1.531)	-0.158<br>(-0.419,0.104)	-0.171<br>(-0.416,0.075)	-0.119<br>(-0.420,0.181)	0.966***<br>(0.493,1.438)
Female				-0.186<br>(-1.231,0.859)	-0.179<br>(-1.188,0.829)	-0.028<br>(-1.026,0.969)
Education (Base: Elementary)						
College Degree				0.604***<br>(0.405,0.803)	0.589***<br>(0.422,0.757)	0.489***<br>(0.329,0.649)
Secondary Education				0.348***<br>(0.165,0.532)	0.343***<br>(0.189,0.498)	0.289***<br>(0.139,0.440)
Unreported				0.634***<br>(0.292,0.976)	0.615***<br>(0.288,0.942)	0.443**<br>(0.156,0.729)
Employment Status (Base: Employed Full Time)						
Employed Full Time for Self				-0.040<br>(-0.155,0.074)	-0.053<br>(-0.167,0.061)	-0.010<br>(-0.107,0.088)
Employed Part Time (do not want full time)				0.168**<br>(0.063,0.273)	0.167***<br>(0.068,0.266)	0.134**<br>(0.040,0.228)
Employed Part Time (want full time)				-0.112<br>(-0.226,0.001)	-0.112*<br>(-0.221,-0.004)	-0.161**<br>(-0.263,-0.059)
Unemployed				-0.607***<br>(-0.767,-0.448)	-0.548***<br>(-0.675,-0.422)	-0.575***<br>(-0.701,-0.450)
Out of Workforce				-0.142**<br>(-0.246,-0.039)	-0.101*<br>(-0.195,-0.008)	-0.083<br>(-0.180,0.014)
Health Problems (Base: Yes)						
No				0.563***<br>(0.458,0.668)	0.569***<br>(0.468,0.670)	0.546***<br>(0.451,0.642)
Unreported				0.149<br>(-0.166,0.463)	0.172<br>(-0.143,0.488)	0.157<br>(-0.157,0.471)
Marital Status (Base: Single)						
Married				-0.111<br>(-0.260,0.038)	-0.141*<br>(-0.263,-0.020)	0.034<br>(-0.071,0.140)
Separated				-0.162<br>(-0.333,0.009)	-0.218**<br>(-0.361,-0.076)	-0.182**<br>(-0.319,-0.045)
Divorced				-0.182*<br>(-0.327,-0.037)	-0.217***<br>(-0.344,-0.089)	-0.116<br>(-0.234,0.002)
Widowed				-0.274**<br>(-0.442,-0.106)	-0.293***<br>(-0.438,-0.148)	-0.100<br>(-0.223,0.022)
Domestic Partner				0.413***<br>(0.279,0.546)	0.363***<br>(0.231,0.495)	0.241***<br>(0.120,0.362)
Unreported				0.269<br>(-0.130,0.669)	0.242<br>(-0.169,0.653)	0.290<br>(-0.117,0.697)
Urban Area (Base: Rural Area)						
Small Town				0.100<br>(-0.120,0.320)	0.091<br>(-0.092,0.273)	-0.015<br>(-0.156,0.127)
Large City				0.104<br>(-0.096,0.304)	0.092<br>(-0.073,0.257)	-0.013<br>(-0.144,0.118)
Suburb of a Large City				0.122<br>(-0.114,0.358)	0.129<br>(-0.060,0.318)	0.026<br>(-0.130,0.182)
Unreported				-0.102<br>(-0.452,0.249)	-0.167<br>(-0.509,0.175)	-0.449**<br>(-0.784,-0.113)
Country Level Controls						
Unemployment (% of total labor force)					-0.019<br>(-0.049,0.011)	-0.044**<br>(-0.078,-0.011)
Gini Index (0-100)					0.002<br>(-0.025,0.029)	-0.009<br>(-0.034,0.016)
Ln Health Expenditure Per Capita $1000						0.250<br>(-0.013,0.514)
Urban Population (% of total population)						0.017**<br>(0.006,0.027)
Absolute Redistribution						0.001<br>(-0.022,0.024)
Constant	4.216***<br>(3.903,4.529)	5.053***<br>(4.812,5.295)	4.254***<br>(3.973,4.535)	6.552***<br>(5.566,7.537)	6.598***<br>(5.249,7.948)	6.840***<br>(5.248,8.433)
Observations	97,339	97,339	97,339	97,339	97,339	97,339
R^2	0.121	0.026	0.121	0.152	0.159	0.181

Note: OLS models for the effect of income and income rank on life evaluation (for Survey Years 2023-2024). Column 1 estimates the effect of income; Column 2 estimates the effect of income rank; Column 3 includes both income and income rank. Column 4 adds individual-level controls, including age, gender, and a four-degree polynomial of age interacted with gender. Column 5 introduces country-level controls with low correlation to log GDP per capita, while Column 6 adds country-level controls that are highly correlated with GDP per capita (correlation > 0.7). Country-level controls correspond to the year prior to the survey, or the most recent available year within the previous five. Columns 3 through 6 also include dummy variables for missing indicators. Outliers below the 5 percentile and above the 95 percentile of incomes are excluded. Inference is based on two-sided t-tests with standard errors clustered at the country level; 95% confidence intervals are shown in parentheses. No multiple-comparison adjustments applied. $^{*}p<0.05$; $^{**}p<0.01$; $^{***}p<0.001$.

Supplementary Table 10: Income Effects on Life Evaluation, Specifications with Incremental Controls, Using Per Capita Annual Income (Household Income Divided by Household Size)

	Life Evaluation					
	(1)	(2)	(3)	(4)	(5)	(6)
Log Income $1000	0.539***		0.591***	0.522***	0.522***	0.152
	(0.455,0.623)		(0.488,0.695)	(0.423,0.621)	(0.395,0.650)	(-0.140,0.444)
Income Rank Index (0-1)		0.999***	-0.583***	-0.568***	-0.564***	0.469
		(0.871,1.127)	(-0.847,-0.319)	(-0.827,-0.309)	(-0.889,-0.239)	(-0.330,1.267)
Female				-0.547	-0.563	-0.436
				(-1.581,0.486)	(-1.572,0.446)	(-1.455,0.583)
Education (Base: Elementary)						
College Degree				0.583***	0.586***	0.531***
				(0.411,0.755)	(0.436,0.736)	(0.381,0.680)
Secondary Education				0.304***	0.310***	0.302***
				(0.149,0.459)	(0.171,0.449)	(0.165,0.439)
Unreported				0.604***	0.593***	0.453**
				(0.299,0.909)	(0.298,0.887)	(0.172,0.734)
Employment Status (Base: Employed Full Time)						
Employed Full Time for Self				-0.012	-0.034	-0.018
				(-0.134,0.110)	(-0.151,0.082)	(-0.118,0.081)
Employed Part Time (do not want full time)				0.170***	0.162***	0.126**
				(0.074,0.266)	(0.068,0.255)	(0.041,0.212)
Employed Part Time (want full time)				-0.106	-0.114*	-0.167**
				(-0.218,0.006)	(-0.221,-0.008)	(-0.268,-0.065)
Unemployed				-0.590***	-0.542***	-0.587***
				(-0.736,-0.444)	(-0.660,-0.423)	(-0.704,-0.470)
Out of Workforce				-0.133**	-0.098*	-0.101*
				(-0.229,-0.037)	(-0.187,-0.009)	(-0.194,-0.008)
Health Problems (Base: Yes)						
No				0.569***	0.575***	0.562***
				(0.458,0.679)	(0.469,0.681)	(0.462,0.661)
Unreported				0.078	0.099	0.118
				(-0.246,0.402)	(-0.230,0.429)	(-0.198,0.433)
Marital Status (Base: Single)						
Married				0.077	0.051	0.171**
				(-0.073,0.226)	(-0.071,0.173)	(0.055,0.287)
Separated				-0.157	-0.213**	-0.174*
				(-0.330,0.016)	(-0.359,-0.068)	(-0.312,-0.036)
Divorced				-0.212**	-0.238***	-0.169**
				(-0.359,-0.064)	(-0.368,-0.108)	(-0.292,-0.046)
Widowed				-0.236**	-0.254***	-0.117
				(-0.401,-0.072)	(-0.397,-0.112)	(-0.244,0.010)
Domestic Partner				0.503***	0.457***	0.357***
				(0.373,0.633)	(0.328,0.586)	(0.233,0.481)
Unreported				0.262	0.241	0.289
				(-0.145,0.669)	(-0.174,0.656)	(-0.126,0.704)
Urban Area (Base: Rural Area)						
Small Town				0.089	0.087	-0.014
				(-0.112,0.291)	(-0.087,0.260)	(-0.152,0.124)
Large City				0.111	0.098	-0.022
				(-0.075,0.297)	(-0.060,0.256)	(-0.152,0.109)
Suburb of a Large City				0.139	0.141	0.031
				(-0.075,0.352)	(-0.032,0.314)	(-0.120,0.183)
Unreported				-0.148	-0.204	-0.440**
				(-0.474,0.179)	(-0.532,0.125)	(-0.766,-0.114)
Country Level Controls						
Unemployment (% of total labor force)					-0.021	-0.042*
					(-0.051,0.009)	(-0.076,-0.007)
Gini Index (0-100)					0.007	-0.006
					(-0.020,0.035)	(-0.031,0.020)
Ln Health Expenditure Per Capita $1000						0.175
						(-0.199,0.550)
Urban Population (% of total population)						0.017**
						(0.007,0.027)
Absolute Redistribution						0.002
						(-0.022,0.025)
Constant	5.092***	5.247***	5.320***	8.565***	8.428***	7.930***
	(4.886,5.299)	(5.009,5.485)	(5.151,5.489)	(7.623,9.508)	(7.164,9.693)	(6.427,9.432)
Observations	97,339	97,339	97,339	97,339	97,339	97,339
R^2	0.116	0.013	0.119	0.153	0.159	0.176

Note: OLS models for the effect of income and income rank on life evaluation (for Survey Years 2023-2024). Column 1 estimates the effect of income; Column 2 estimates the effect of income rank; Column 3 includes both income and income rank. Column 4 adds individual-level controls, including age, gender, and a four-degree polynomial of age interacted with gender. Column 5 introduces country-level controls with low correlation to log GDP per capita, while Column 6 adds country-level controls that are highly correlated with GDP per capita (correlation > 0.7). Income is measured as per capita annual household income, based on Gallup World Poll data, calculated by dividing total annual household income by the number of household members. Country-level controls correspond to the year prior to the survey, or the most recent available year within the previous five. Columns 3 through 6 also include dummy variables for missing indicators. Outliers below the 5 percentile and above the 95 percentile of incomes are excluded. Inference is based on two-sided t-tests with standard errors clustered at the country level; 95% confidence intervals are shown in parentheses. No multiple-comparison adjustments applied. *p<0.05; **p<0.01; ***p<0.001.

Supplementary Table 11: Income Effects on Life Evaluation, High vs. Low GDP per Capita Countries

	Life Evaluation					
	Below Median GDP Per Capita			Above Median GDP Per Capita		
	(1)	(2)	(3)	(4)	(5)	(6)
Log Income $1000	0.328***		0.077	0.304***		-0.192
	(0.217,0.438)		(-0.159,0.314)	(0.138,0.470)		(-0.460,0.077)
Income Rank Index (0-1)		1.182***	0.988**		1.038***	1.394***
		(0.943,1.420)	(0.321,1.654)		(0.904,1.172)	(0.862,1.927)
Female	-1.010	-0.907	-0.919	0.636	0.728	0.764
	(-2.473,0.453)	(-2.351,0.537)	(-2.366,0.528)	(-0.741,2.012)	(-0.634,2.090)	(-0.608,2.137)
Education (Base: Elementary)						
College Degree	0.623***	0.548***	0.548***	0.399***	0.378***	0.414***
	(0.378,0.868)	(0.286,0.809)	(0.287,0.810)	(0.242,0.557)	(0.232,0.525)	(0.267,0.562)
Secondary Education	0.365***	0.331**	0.332***	0.158*	0.171**	0.200**
	(0.183,0.547)	(0.133,0.529)	(0.135,0.528)	(0.023,0.292)	(0.041,0.301)	(0.074,0.325)
Unreported	0.407	0.375	0.380	0.364*	0.385*	0.403**
	(-0.071,0.884)	(-0.098,0.848)	(-0.097,0.857)	(0.061,0.666)	(0.092,0.678)	(0.113,0.693)
Employment Status (Base: Employed Full Time)						
Employed Full Time for Self	-0.115	-0.101	-0.100	0.135**	0.128**	0.119**
	(-0.249,0.018)	(-0.232,0.031)	(-0.231,0.031)	(0.053,0.216)	(0.045,0.211)	(0.038,0.200)
Employed Part Time (do not want full time)	0.036	0.021	0.030	0.237***	0.249***	0.238***
	(-0.120,0.192)	(-0.148,0.190)	(-0.125,0.185)	(0.132,0.342)	(0.139,0.358)	(0.131,0.344)
Employed Part Time (want full time)	-0.248***	-0.267***	-0.255***	-0.021	-0.018	-0.046
	(-0.376,-0.119)	(-0.414,-0.119)	(-0.387,-0.123)	(-0.136,0.094)	(-0.135,0.098)	(-0.157,0.064)
Unemployed	-0.711***	-0.703***	-0.695***	-0.404***	-0.358***	-0.370***
	(-0.885,-0.537)	(-0.884,-0.521)	(-0.869,-0.522)	(-0.536,-0.273)	(-0.483,-0.233)	(-0.498,-0.243)
Out of Workforce	-0.173*	-0.151	-0.151	-0.069	-0.034	-0.039
	(-0.326,-0.020)	(-0.312,0.011)	(-0.311,0.009)	(-0.169,0.032)	(-0.129,0.060)	(-0.134,0.055)
Health Problems (Base: Yes)						
No	0.403***	0.395***	0.392***	0.734***	0.714***	0.718***
	(0.242,0.563)	(0.243,0.548)	(0.239,0.546)	(0.654,0.814)	(0.635,0.794)	(0.637,0.798)
Unreported	0.244	0.226	0.235	0.102	0.118	0.123
	(-0.245,0.732)	(-0.258,0.710)	(-0.248,0.717)	(-0.297,0.500)	(-0.284,0.521)	(-0.284,0.530)
Marital Status (Base: Single)						
Married	-0.132	-0.108	-0.116	0.178***	0.165***	0.187***
	(-0.315,0.051)	(-0.291,0.075)	(-0.296,0.063)	(0.101,0.255)	(0.085,0.245)	(0.110,0.264)
Separated	-0.255*	-0.270**	-0.266*	-0.111	-0.128	-0.139
	(-0.454,-0.056)	(-0.472,-0.069)	(-0.469,-0.063)	(-0.273,0.050)	(-0.285,0.028)	(-0.291,0.014)
Divorced	-0.272*	-0.261*	-0.260*	-0.055	-0.016	-0.005
	(-0.539,-0.005)	(-0.511,-0.011)	(-0.513,-0.008)	(-0.150,0.041)	(-0.107,0.075)	(-0.096,0.086)
Widowed	-0.331**	-0.291**	-0.298**	-0.025	0.001	0.009
	(-0.544,-0.118)	(-0.504,-0.077)	(-0.507,-0.088)	(-0.146,0.096)	(-0.118,0.120)	(-0.108,0.127)
Domestic Partner	0.287**	0.240**	0.250**	0.268***	0.230***	0.232***
	(0.109,0.466)	(0.062,0.419)	(0.071,0.430)	(0.117,0.419)	(0.098,0.363)	(0.102,0.361)
Unreported	0.964*	0.950*	0.952*	-0.008	0.044	0.057
	(0.114,1.814)	(0.101,1.799)	(0.101,1.802)	(-0.345,0.330)	(-0.291,0.379)	(-0.276,0.390)
Urban Area (Base: Rural Area)						
Small Town	0.038	0.028	0.028	-0.066	-0.059	-0.049
	(-0.158,0.234)	(-0.170,0.226)	(-0.170,0.226)	(-0.178,0.046)	(-0.168,0.050)	(-0.157,0.059)
Large City	-0.064	-0.069	-0.076	-0.039	-0.010	0.016
	(-0.253,0.126)	(-0.263,0.125)	(-0.267,0.116)	(-0.181,0.102)	(-0.149,0.128)	(-0.117,0.150)
Suburb of a Large City	0.052	0.059	0.050	-0.003	0.018	0.042
	(-0.168,0.272)	(-0.166,0.285)	(-0.168,0.268)	(-0.175,0.170)	(-0.146,0.182)	(-0.122,0.205)
Unreported	1.540***	1.652***	1.649***	-0.464**	-0.420*	-0.392*
	(0.884,2.196)	(0.987,2.317)	(0.986,2.312)	(-0.791,-0.138)	(-0.747,-0.094)	(-0.730,-0.055)
Country Level Controls						
Ln Health Expenditure Per Capita $1000	0.006	0.196	0.153	0.037	0.225	0.339*
	(-0.307,0.320)	(-0.100,0.492)	(-0.213,0.518)	(-0.284,0.358)	(-0.047,0.497)	(0.048,0.630)
Unemployment (% of total labor force)	-0.052	-0.058	-0.057	-0.028	-0.029	-0.030
	(-0.111,0.007)	(-0.116,0.001)	(-0.115,0.002)	(-0.069,0.013)	(-0.067,0.008)	(-0.066,0.007)
Urban Population (% of total population)	0.021**	0.022**	0.022**	0.008	0.010*	0.012*
	(0.006,0.036)	(0.006,0.038)	(0.006,0.037)	(-0.002,0.018)	(0.001,0.020)	(0.002,0.022)
Gini Index (0-100)	-0.011	-0.029	-0.025	0.025	0.020	0.017
	(-0.047,0.024)	(-0.062,0.003)	(-0.059,0.009)	(-0.002,0.053)	(-0.008,0.047)	(-0.012,0.046)
Absolute Redistribution	-0.003	-0.008	-0.007	0.030	0.036*	0.040*
	(-0.059,0.053)	(-0.072,0.057)	(-0.069,0.055)	(-0.006,0.066)	(0.002,0.069)	(0.007,0.073)
Constant	7.539***	8.293***	8.060***	5.300***	5.079***	5.279***
	(5.234,9.845)	(6.149,10.437)	(5.761,10.359)	(3.531,7.068)	(3.243,6.915)	(3.451,7.106)
Observations	48,155	48,155	48,155	49,184	49,184	49,184
R^2	0.111	0.114	0.114	0.101	0.111	0.112

Note: OLS models for the effect of income and income rank on life evaluation (for Survey Years 2023-2024). The sample is split by median GDP per capita: columns 1 to 3 present results for countries below the median, and columns 4 to 6 for those above. All models include individual level controls (including age, gender, a fourth-degree polynomial in age, and interactions between age and gender) as well as country level controls. Country level controls are taken from the year prior to the survey, or the most recent available year within the past five. Dummy variables are included for any missing country level data. Outliers below the 5 percentile and above the 95 percentile of incomes are excluded. Inference is based on two-sided t-tests with standard errors clustered at the country level; 95% confidence intervals are shown in parentheses. No multiple-comparison adjustments applied. *p<0.05; **p<0.01; ***p<0.001.

Supplementary Table 12: BIC for the Generalized Rank and Relative Rank Models (part 1)

	**Round 17: 2022-2023**				**Round 18: 2023-2024**			
	$\delta \geq 0$ $\gamma = 0$	$\delta = 1$ $-10 \leq \gamma \leq 10$	$\delta = 1$ $-10 \leq \gamma_d \leq 10$ $-10 \leq \gamma_u \leq 10$	$\delta = 1$ $\gamma = 0$	$\delta \geq 0$ $\gamma = 0$	$\delta = 1$ $-10 \leq \gamma \leq 10$	$\delta = 1$ $-10 \leq \gamma_d \leq 10$ $-10 \leq \gamma_u \leq 10$	$\delta = 1$ $\gamma = 0$
	(1)	(2)	(3)	(4)	(5)	(6)	(7)	(8)
Afghanistan	2214.136	2213.332	2219.742	2207.391	2727.393	2739.069	**2725.715**	2761.777
Albania	4041.737	4045.501	4046.853	4041.002	4031.406	4030.371		4025.153
Armenia	4032.907	4032.091	4039.493	4026.118	3911.874	3916.18		3909.415
Australia	3323.552	3323.193	3338.62	3318.072	3542.856	3542.845	3559.052	3536.142
Azerbaijan	4029.276	4028.134	4034.529	4022.362	3564.67	3566.415	3570.084	3558.412
Bangladesh	3680.256	3681.007	3687.489	3671.914	**3922.017**	3924.986	3928.946	3922.144
Belgium	3200.677	3201.929	3207.395	3195.564	3272.444	3271.47	3304.382	3265.444
Bosnia and Herzegovina	3744.532	3746.675	3756.941	3738.658	3723.342	3728.402		3720.467
Botswana	3323.287	3323.461	3329.795	3316.583	3998.985	3998.583	4004.585	3991.161
Bulgaria	3640.189	3638.4	3651.124	3634.505	**3599.599**	3614.64	3647.755	3607.338
Cambodia	3688.132	3684.623	3686.434	3681.932	4221.193	4221.475	4228.112	4219.034
Cameroon	4268.94	4269.332	4276.078	4265.988	4363.738	4363.557	4367.209	4356.66
Canada	3563.96	3563.757		3557.642	3620.933	3625.854		3619.111
Chile	3611.63	3612.736	3621.314	3606.32	3853.134	3853.655		3846.447
Colombia	4153.749	4153.741	4160.715	4146.574	3939.972	3946.275	3983.624	3939.608
Costa Rica	3823.675	3823.798	3835.089	3817.957	3812.815	3813.246		3806.684
Croatia	3510.07	**3502.6**	3509.002	3502.81	3505.887	3508.372	3505.097	3504.578
Cyprus	3633.886	3632.276		3627.149	3755.642	3759.136	3761.911	3753.39
Czech Republic	3582.347	3582.591		3575.946	3533.874	3532.928	3539.38	3527.392
Denmark	3218.255	3215.271	3258.314	3211.614	3395.881	3397.025		3391.764
Dominican Republic	4465.618	4465.481		4459.333	4497.676	4496.625		4494.037
Ecuador	3963.125	3964.68		3958.705	**4110.867**	4118.894	4121.099	4112.37
Egypt	3631.163	3626.086	3632.854	3624.099	3668.761	3673.584	3676.518	3665.354
El Salvador	3862.156	3861.637		3856.117	3789.477	3788.994		3783.785
Estonia	3221.601	3222.351	3225.887	3215.687	3238.959	3244.576	3247.278	3238.249
Ethiopia	4034.572	4035.85	4042.603	4029.231	4031.975	4036.352	4040.632	4029.45
Finland	2951.217	**2945.325**	2950.416	2946.243	2996.553	2998.42	3003.939	2992.091
France	3665.829	3667.438	3672.439	3660.751	3560.73	3558.756	3565.54	3553.92
Gabon	4138.385	4136.988		4131.767	4121.966	4122.213		4115.64
Georgia	3836.124	3835.586	3841.923	3830.329	3747.884	3747.182	3760.287	3741.562
Germany	3504.232	3506.579	3533.17	3502.092	3548.706	3553.139	3554.364	3547.313
Greece	**3366.172**	3373.807	3373.717	3368.098	**3480.24**	3491.538	3487.737	3487.615
Guatemala	3970.527	3970.335	3983.074	3964.554	3787.832	3788.549	3790.59	3782.124
Honduras	3813.164	3812.619		3806.57	3587.986	3587.924		3581.006
Hungary	3472.901	3471.368	3477.936	3466.549	3686.242	3686.663	3693.009	3680.4
India	12446.729	12443.762	12450.711	12440.799	12087.523	12087.767	12091.902	12082.739
Indonesia	3643.468	3642.616		3637.904	3966.878	3965.754		3960.677
Iraq	4421.715	4420.683	4456.338	4414.638	**4371.536**	4378.286		4373.95
Israel	2933.309	2933.433	2940.003	2928.914	**3681.74**	3690.72		3687.868
Italy	3571.161	3571.125		3564.387	3606.119	3608.409	3612.978	3601.986
Ivory Coast	4237.525	4240.15	4246.302	4236.272	4168.752	4170.734	4172.875	4164.179
Japan	3716.01	3717.195	3722.674	3710.185	3631.514	3632.376		3625.726
Jordan	4366.264	4365.697	4372.284	4359.369	4338.487	4338.468	4344.827	4331.645
Kazakhstan	3577.631	3575.699		3570.652	3573.939	3571.402		3570.997
Kosovo	3940.475	3940.821	3947.302	3933.686	3812.938	3811.703	3818.319	3806.239
Kyrgyzstan	3632.419	3628.149	3633.372	3625.613	3688.579	3687.571		3682.203
Latvia	3349.856	3347.889	3354.642	3342.546	3320.149	3320.62	3326.73	3313.689
Lithuania	**3107.459**	3130.134	3110.221	3120.793	3323.839	3328.648	3329.471	3321.572
Mali	4241.777	**4236.134**		4237.077	4110.213	4110.407	4126.14	4104.511
Malta	3623.256	3623.352	3629.84	3616.516	3706.89	3706.28		3700.656
Mexico	3844.756	3846.582	3845.395	3839.305	3948.79	3947.613		3943.736

Note: Bayesian information criterion (BIC) values of the four versions of generalized rank: the generalized rank model that allows for asymmetry in upward and downward comparisons (Columns 1 and 5), the generalized rank model that allows for greater weights to those peers closer (or farther away) (Columns 2 and 6, assuming γ is identical for upward and downward comparisons; and Columns 3 and 7, allowing γ_u for upward comparisons to be different from that of downward comparisons γ_d), and the relative rank model (Columns 4 and 8). The BIC shows that the relative rank model better fits the data except for the cases shown in bold. Countries shown are those for which δ and γ could be estimated in both survey rounds, providing a consistent baseline sample across parameters.

Supplementary Table 12: BIC for the Generalized Rank and Relative Rank Models (part 2)

	Round 17: 2022-2023				Round 18: 2023-2024			
	$\delta \geq 0$<br>$\gamma = 0$	$\delta = 1$<br>$-10 \leq \gamma \leq 10$	$\delta = 1$<br>$-10 \leq \gamma_d \leq 10$<br>$-10 \leq \gamma_u \leq 10$	$\delta = 1$<br>$\gamma = 0$	$\delta \geq 0$<br>$\gamma = 0$	$\delta = 1$<br>$-10 \leq \gamma \leq 10$	$\delta = 1$<br>$-10 \leq \gamma_d \leq 10$<br>$-10 \leq \gamma_u \leq 10$	$\delta = 1$<br>$\gamma = 0$
	(1)	(2)	(3)	(4)	(5)	(6)	(7)	(8)
Moldova	3799.312	3797.368		3792.587	3390.04	3390.144	3420.028	3382.648
Montenegro	3536.231	3535.209	3537.798	3531.763	3411.654	3414.398	3417.36	3405.443
Morocco	3833.89	3833.932	3836.263	3827.814	3856.632	3856.622	3863.322	3848.746
Myanmar	3808.136	3808.044	3814.823	3800.631	3828.111	3831.249	3836.407	3824.756
Netherlands	2925.122	2926.327	2931.243	2921.304	3137.495	3136.825	3143.617	3131.035
New Zealand	3418.808	3421.588	3432.632	3414.614	3507.563	3512.705		3502.109
Nicaragua	4370.269	4370.372	4376.291	4364.448	4219.778	4220.281		4213.427
North Macedonia	3934.505	3934.762	3941.292	3928.831	3547.892	3548.598	3554.712	3542.119
Pakistan	4108.952	4112.478	4106.802	4105.546	4079.614	4081.091	4086.174	4075.975
Palestinian Territories	3892.985	3907.028	**3891.121**	3900.348	4029.76	4034.501	4038.276	4028.398
Panama	3853.584	3854.83		3848.906	3824.828	3825.735		3820.742
Paraguay	4025.627	4025.103		4018.275	4151.335	4151.577	4157.967	4144.544
Peru	4038.501	4040.507		4035.187	4127.509	4128.133		4121.048
Philippines	4100.118	4100.331	4105.564	4093.753	4132.702	4134.385		4128.493
Poland	3176.92	3176.947	3183.635	3169.883	3203.354	3205.658		3198.863
Portugal	3739.459	3739.749	3746.133	3732.958	3632.706	3632.566	3639.31	3625.556
Romania	3640.708	3641.906		3635.634	3789.696	3790.35	3803.856	3783.438
Senegal	4241.445	4241.736		4234.665	4093.2	4093.162	4099.102	4086.679
Serbia	**3772.371**	3783.666	3780.242	3777.688	**3721.788**	3723.994	3726.359	3721.828
Slovakia	3554.397	3552.708	3556.068	3550.123	3406.33	3406.404		3399.684
Slovenia	3388.289	3386.958	3393.009	3381.625	3510.388	3514.463	3517.604	3509.491
South Africa	4053.026	4052.053		4045.691	4115.158	4114.473	4132.275	4110.708
South Korea	3616.058	3613.076	3616.052	3610.258	3681.572	3679.793	3686.19	3673.995
Spain	3670.761	3671.575		3664.493	3558.91	3559.232	3565.47	3552.461
Sri Lanka	4068.149	4065.55	4072.534	4063.064	3992.77	3993.389	3999.468	3986.317
Sweden	3122.409	3121.033	3127.416	3114.9	3296.556	3295.921	3302.094	3289.928
Tajikistan	3312.757	3314.463	3320.505	3306.986	3414.749	3418.685	3422.515	3410.019
Tanzania	4321.656	4317.705	4323.204	4317.344	4025.075	4026.011	4032.191	4019.553
Tunisia	3628.305	3631.79	3634.369	3625.736	3461.657	3461.58	3468.203	3454.351
Turkey	3878.18	3878.198	3884.982	3871.616	3706.61	3706.678		3701.03
Uganda	4342.621	4342.723	4349.424	4336.208	3802.454	3802.541	3802.267	3795.929
Ukraine	3958.079	3956.549	3962.902	3952.075	3787.385	3786.614	3792.911	3780.1
United Arab Emirates	4034.964	4037.067		4033.3	3707.758	3711.818		3707.579
United States	3646.256	3646.619	3652.427	3639.752	3835.128	3837.791	3841.962	3830.961
Uruguay	3893.881	3892.399	3908.941	3886.959	3889.533	3889.501		3882.648
Vietnam	3039.011	3041.228	3045.953	3034.934	**3552.201**	3568.698	3559.093	3560.608
Zambia	4287.119	4286.43	4293.132	4280.548	4260.618	4259.669	4265.86	4253.146

Note: Bayesian information criterion (BIC) values of the four versions of generalized rank: the generalized rank model that allows for asymmetry in upward and downward comparisons (Columns 1 and 5), the generalized rank model that allows for greater weights to those peers closer (or farther away) (Columns 2 and 6, assuming γ is identical for upward and downward comparisons; and Columns 3 and 7, allowing γ_u for upward comparisons to be different from that of downward comparisons γ_d), and the relative rank model (Columns 4 and 8). The BIC shows that the relative rank model better fits the data except for the cases shown in bold. Countries shown are those for which δ and γ could be estimated in both survey rounds, providing a consistent baseline sample across parameters.

Supplementary Table 13: Country Level Moderators of the Income Rank Effect - Economic Development and Inequality

	(1)	(2)	(3)
Income Rank Index (0-1)	1.123***	0.854*	1.392***
	(0.682,1.565)	(0.194,1.513)	(0.972,1.811)
GDP			
Ln GDP per capita, PPP (2011) $1000	0.331		
	(-0.406,1.069)		
Ln GDP per capita, PPP (2011) $1000 × Income Rank Index	0.001		
	(-0.125,0.127)		
Inequiality			
Gini Index (0-100)		-0.015	
		(-0.043,0.014)	
Gini Index (0-100) × Income Rank Index		0.008	
		(-0.012,0.027)	
Absolute Redistribution			0.020
			(-0.017,0.056)
Absolute Redistribution × Income Rank Index			-0.018
			(-0.042,0.005)
Constant	6.100***	6.777***	5.530***
	(3.289,8.911)	(5.218,8.336)	(3.637,7.423)
Individual Level Controls	YES	YES	YES
Country Level Controls	YES	YES	YES
Observations	97,339	80,752	52,961
R^2	0.182	0.170	0.141

Note: OLS regression estimates of the interaction effect of income rank and country level indicators. All columns include the full set of individual and country level controls of the baseline model. However, to ease interpretation, only the specific level and interaction terms tested on each column are displayed. Reliable measures of absolute redistribution are only available for 58 countries. Inference is based on two-sided t-tests with standard errors clustered at the country level; 95% confidence intervals are shown in parentheses. No multiple-comparison adjustments applied. $^{*}p<0.05$; $^{**}p<0.01$; $^{***}p<0.001$.

Supplementary Table 14: Country Level Moderators of the Income Rank Effect - Economic Competitiveness

	(1)	(2)	(3)	(4)	(5)	(6)
Income Rank Index (0-1)	1.116***	1.115***	1.126***	1.120***	1.114***	1.118***
	(0.965,1.267)	(0.961,1.270)	(0.976,1.276)	(0.971,1.269)	(0.960,1.269)	(0.969,1.268)
Institutions						
Institutions	-0.077					
	(-0.288,0.134)					
Institutions × Income Rank Index	-0.001					
	(-0.131,0.128)					
Higher Education						
Higher Education		-0.047				
		(-0.355,0.262)				
Higher Education × Income Rank Index		0.029				
		(-0.122,0.181)				
Goods Market						
Goods Market			0.161			
			(-0.057,0.378)			
Goods Market × Income Rank Index			-0.081			
			(-0.231,0.070)			
Labor Market						
Labor Market				0.018		
				(-0.184,0.220)		
Labor Market × Income Rank Index				-0.041		
				(-0.184,0.102)		
Business Sophistication						
Business Sophistication					0.003	
					(-0.218,0.225)	
Business Sophistication × Income Rank Index					0.034	
					(-0.109,0.177)	
Innovation						
Innovation						-0.133
						(-0.321,0.056)
Innovation × Income Rank Index						-0.036
						(-0.147,0.075)
Constant	6.990***	7.056***	7.211***	7.076***	7.063***	6.965***
	(5.563,8.417)	(5.616,8.496)	(5.777,8.645)	(5.638,8.515)	(5.614,8.513)	(5.547,8.382)
Individual Level Controls	YES	YES	YES	YES	YES	YES
Country Level Controls	YES	YES	YES	YES	YES	YES
Observations	91,248	91,248	91,248	91,248	91,248	91,248
R^2	0.175	0.175	0.176	0.175	0.175	0.176

Note: OLS regression estimates of the interaction effect of income rank and country level indicators. All columns include the full set of individual and country level controls of the baseline model. However, to ease interpretation, only the specific level and interaction terms tested on each column are displayed. Inference is based on two-sided t-tests with standard errors clustered at the country level; 95% confidence intervals are shown in parentheses. No multiple-comparison adjustments applied. *p<0.05; **p<0.01; ***p<0.001.

Supplementary Table 15: Country Level Moderators of the Income Rank Effect - Economic Openness

	(1)	(2)	(3)	(4)
Income Rank Index (0-1)	1.094***	1.093***	1.100***	1.105***
	(0.941,1.247)	(0.941,1.246)	(0.949,1.250)	(0.954,1.255)
Market Access Infrastructure				
Market Access Infrastructure	0.179			
	(-0.409,0.766)			
Market Access Infrastructure × Income Rank Index	0.044			
	(-0.087,0.174)			
Investment Environment				
Investment Environment		-0.033		
		(-0.392,0.327)		
Investment Environment × Income Rank Index		0.034		
		(-0.093,0.161)		
Enterprise Conditions				
Enterprise Conditions			-0.105	
			(-0.325,0.114)	
Enterprise Conditions × Income Rank Index			-0.008	
			(-0.140,0.124)	
Governance				
Governance				0.011
				(-0.238,0.260)
Governance × Income Rank Index				-0.030
				(-0.148,0.087)
Constant	6.991***	6.974***	6.870***	7.002***
	(5.483,8.499)	(5.532,8.416)	(5.408,8.332)	(5.540,8.463)
Individual Level Controls	YES	YES	YES	YES
Country Level Controls	YES	YES	YES	YES
Observations	95,596	95,596	95,596	95,596
R^2	0.180	0.180	0.180	0.180

Note: OLS regression estimates of the interaction effect of income rank and country level indicators. All columns include the full set of individual and country level controls of the baseline model. However, to ease interpretation, only the specific level and interaction terms tested on each column are displayed. Inference is based on two-sided t-tests with standard errors clustered at the country level; 95% confidence intervals are shown in parentheses. No multiple-comparison adjustments applied. *p<0.05; **p<0.01; ***p<0.001.

Supplementary Table 16: Country Level Moderators of the Income Rank Effect - Health Spending, Employment, and Population Dynamics

	(1)	(2)	(3)
Income Rank Index (0-1)	1.098***	0.861***	1.308***
	(0.947,1.249)	(0.635,1.087)	(0.808,1.808)
Health Expenditure			
Ln Health Expenditure Per Capita, PPP (2011) $1000	0.286**		
	(0.072,0.500)		
Ln Health Expenditure Per Capita, PPP (2011) $1000 × Income Rank Index	0.018		
	(-0.088,0.123)		
Unemployment			
Unemployment (% of total labor force)		-0.071**	
		(-0.114,-0.028)	
Unemployment (% of total labor force) × Income Rank Index		0.044**	
		(0.017,0.071)	
Urban Population			
Urban Population (% of total population)			0.018**
			(0.007,0.030)
Urban Population (% of total population) × Income Rank Index			-0.003
			(-0.010,0.004)
Constant	6.988***	6.889***	6.914***
	(5.460,8.516)	(5.257,8.520)	(5.343,8.486)
Individual Level Controls	YES	YES	YES
Country Level Controls	YES	YES	YES
Observations	95,596	93,024	96,475
R^2	0.180	0.188	0.180

Note: OLS regression estimates of the interaction effect of income rank and country level indicators. All columns include the full set of individual and country level controls of the baseline model. However, to ease interpretation, only the specific level and interaction terms tested on each column are displayed. Inference is based on two-sided *t*-tests with standard errors clustered at the country level; 95% confidence intervals are shown in parentheses. No multiple-comparison adjustments applied. *p<0.05; **p<0.01; ***p<0.001.

Supplementary Table 17: Country Level Moderators of the Income Rank Effect - Confidence and Trust in Society and Institutions

	(1)	(2)	(3)	(4)	(5)	(6)	(7)
Income Rank Index (0-1)	1.118***	1.151***	1.123***	1.127***	1.134***	1.120***	1.112***
	(0.977,1.259)	(1.002,1.299)	(0.976,1.271)	(0.989,1.265)	(0.985,1.284)	(0.977,1.264)	(0.975,1.249)
Law and Order Index							
Law and Order Index	0.036						
	(-0.133,0.205)						
Law and Order Index × Income Rank Index	0.066						
	(-0.088,0.221)						
National Institutions Index							
National Institutions Index		0.079					
		(-0.115,0.272)					
National Institutions Index × Income Rank Index		-0.070					
		(-0.264,0.123)					
Corruption Index							
Corruption Index			-0.119				
			(-0.274,0.036)				
Corruption Index × Income Rank Index			0.139**				
			(0.033,0.244)				
Social Life Index							
Social Life Index				0.555***			
				(0.362,0.748)			
Social Life Index × Income Rank Index				-0.105			
				(-0.245,0.035)			
Civic Engagement Index							
Civic Engagement Index					0.258**		
					(0.092,0.424)		
Civic Engagement Index × Income Rank Index					-0.223***		
					(-0.351,-0.095)		
Community Basics Index							
Community Basics Index						0.282**	
						(0.084,0.480)	
Community Basics Index × Income Rank Index						-0.043	
						(-0.183,0.096)	
Migrant Acceptance Index							
Migrant Acceptance Index							0.214**
							(0.076,0.352)
Migrant Acceptance Index × Income Rank Index							-0.248***
							(-0.373,-0.123)
Constant	6.784***	7.543***	6.887***	6.568***	7.262***	6.783***	7.156***
	(5.306,8.262)	(6.164,8.922)	(5.252,8.522)	(5.336,7.800)	(5.681,8.842)	(5.370,8.196)	(5.668,8.645)
Individual Level Controls	YES	YES	YES	YES	YES	YES	YES
Country Level Controls	YES	YES	YES	YES	YES	YES	YES
Observations	97,339	86,732	87,694	97,339	97,339	97,339	97,339
R^2	0.181	0.164	0.180	0.204	0.184	0.189	0.182

Note: OLS regression estimates of the interaction effect of income rank and country level indicators. All columns include the full set of individual and country level controls of the baseline model. However, to ease interpretation, only the specific level and interaction terms tested on each column are displayed. Inference is based on two-sided *t*-tests with standard errors clustered at the country level; 95% confidence intervals are shown in parentheses. No multiple-comparison adjustments applied. *p<0.05; **p<0.01; ***p<0.001.

Supplementary Table 18: Country Level Moderators of the Income Rank Effect - Materialism, Individualism and Autonomy

	(1)	(2)	(3)	(4)	(5)
Income Rank Index (0-1)	1.251***	1.389***	1.268***	1.278***	1.274***
	(1.062,1.440)	(1.139,1.639)	(1.077,1.458)	(1.075,1.481)	(1.078,1.469)
Materialism					
Materialist Index	-0.303***				
	(-0.475,-0.130)				
Materialist Index × Income Rank Index	0.218***				
	(0.095,0.341)				
Importance to be Rich/Successful		-0.166			
		(-0.430,0.098)			
Importance to be Rich/Successful × Income Rank Index		0.107			
		(-0.085,0.299)			
Importance of Work vs Leisure Time			-0.103		
			(-0.408,0.203)		
Importance of Work vs Leisure Time × Income Rank Index			0.099		
			(-0.094,0.291)		
Individualism					
Autonomy Index				0.023	
				(-0.310,0.356)	
Autonomy Index × Income Rank Index				-0.123	
				(-0.319,0.073)	
Importance of Friends vs Family					0.137
					(-0.039,0.313)
Importance of Friends vs Family × Income Rank Index					-0.130
					(-0.289,0.029)
Constant	7.723***	6.349***	7.077***	7.114***	7.058***
	(5.712,9.734)	(3.984,8.714)	(4.969,9.185)	(4.883,9.344)	(4.913,9.202)
Individual Level Controls	YES	YES	YES	YES	YES
Country Level Controls	YES	YES	YES	YES	YES
Observations	46,194	40,302	46,194	46,194	46,194
R^2	0.164	0.174	0.160	0.160	0.160

Note: OLS regression estimates of the interaction effect of income rank and country level indicators. All columns include the full set of individual and country level controls of the baseline model. However, to ease interpretation, only the specific level and interaction terms tested on each column are displayed. The Materialist Index corresponds to the reverse-coded 12-item Inglehart post-materialism score. The other two measures of materialism reflect the country-level importance of wealth and success, and the relative importance of work versus leisure. Inference is based on two-sided t-tests with standard errors clustered at the country level; 95% confidence intervals are shown in parentheses. No multiple-comparison adjustments applied. *p<0.05; **p<0.01; ***p<0.001.

Supplementary Table 19: Country Level Moderators of the Income Rank Effect - Social Preferences

	(1)	(2)	(3)	(4)	(5)	(6)
Income Rank Index (0-1)	1.192***	1.179***	1.209***	1.181***	1.207***	1.188***
	(1.022,1.362)	(1.014,1.343)	(1.038,1.380)	(1.001,1.361)	(1.038,1.377)	(1.018,1.359)
Patience						
Patience	0.028					
	(-0.182,0.238)					
Patience × Income Rank Index	-0.109					
	(-0.231,0.013)					
Risk Taking						
Risk Taking		0.015				
		(-0.219,0.249)				
Risk Taking × Income Rank Index		-0.079				
		(-0.253,0.096)				
Positive Reciprocity						
Positive Reciprocity			-0.174			
			(-0.373,0.025)			
Positive Reciprocity × Income Rank Index			-0.020			
			(-0.203,0.163)			
Negative Reciprocity						
Negative Reciprocity				-0.093		
				(-0.280,0.093)		
Negative Reciprocity × Income Rank Index				0.008		
				(-0.117,0.133)		
Altruism						
Altruism					-0.199*	
					(-0.370,-0.028)	
Altruism × Income Rank Index					-0.019	
					(-0.151,0.114)	
Trust						
Trust						-0.001
						(-0.266,0.264)
Trust × Income Rank Index						-0.021
						(-0.168,0.127)
Constant	6.310***	6.222***	6.470***	6.402***	6.370***	6.276***
	(4.094,8.525)	(3.963,8.481)	(4.308,8.633)	(4.262,8.542)	(4.204,8.535)	(4.096,8.456)
Individual Level Controls	YES	YES	YES	YES	YES	YES
Country Level Controls	YES	YES	YES	YES	YES	YES
Observations	59,240	59,240	59,240	59,240	59,240	59,240
R^2	0.208	0.208	0.212	0.209	0.213	0.207

Note: OLS regression estimates of the interaction effect of income rank and country level indicators. All columns include the full set of individual and country level controls of the baseline model. However, to ease interpretation, only the specific level and interaction terms tested on each column are displayed. Inference is based on two-sided t-tests with standard errors clustered at the country level; 95% confidence intervals are shown in parentheses. No multiple-comparison adjustments applied. *p<0.05; **p<0.01; ***p<0.001.

Supplementary Table 20: Individual Level Moderators of the Income Rank Effect - Demographics

	(1)	(2)	(3)	(4)	(5)	(6)
Income Rank Index (0-1)	0.811***	1.098***	1.136***	1.077***	0.895***	1.154***
	(0.614,1.007)	(0.927,1.269)	(0.875,1.396)	(0.868,1.287)	(0.736,1.054)	(0.993,1.315)
Age						
29 to 40 years	-0.467***					
	(-0.578,-0.355)					
40 to 54 years	-0.512***					
	(-0.684,-0.339)					
55 to 99 years	-0.287*					
	(-0.520,-0.055)					
29 to 40 years × Income Rank Index	0.404***					
	(0.264,0.544)					
40 to 54 years × Income Rank Index	0.489***					
	(0.295,0.684)					
55 to 99 years × Income Rank Index	0.422***					
	(0.195,0.648)					
Gender						
Female		0.255***				
		(0.158,0.352)				
Female × Income Rank Index		0.033				
		(-0.098,0.164)				
Education						
College Degree			0.542***			
			(0.333,0.751)			
Secondary Education			0.292**			
			(0.110,0.475)			
College Degree × Income Rank Index			-0.086			
			(-0.357,0.185)			
Secondary Education × Income Rank Index			-0.004			
			(-0.217,0.210)			
Employment						
Employed Part Time (do not want full time)				0.163		
				(-0.015,0.341)		
Employed Part Time (want full time)				-0.021		
				(-0.192,0.150)		
Unemployed				-0.699***		
				(-0.874,-0.525)		
Out of workforce				-0.152		
				(-0.316,0.012)		
Employed Part Time (do not want full time) × Income Rank Index				-0.072		
				(-0.321,0.177)		
Employed Part Time (want full time) × Income Rank Index				-0.321*		
				(-0.577,-0.064)		
Unemployed × Income Rank Index				0.264		
				(-0.004,0.533)		
Out of workforce × Income Rank Index				0.149		
				(-0.041,0.339)		
Marital Status						
Married					-0.111	
					(-0.265,0.044)	
Domestic Partner					0.217*	
					(0.004,0.430)	
Separated/Divorced/Widowed					-0.327***	
					(-0.475,-0.179)	
Married × Income Rank Index					0.302***	
					(0.142,0.463)	
Domestic Partner × Income Rank Index					0.050	
					(-0.212,0.311)	
Separated/Divorced/Widowed × Income Rank Index					0.458***	
					(0.264,0.653)	
Urban/Rural						
Large City/Suburb						0.063
						(-0.064,0.191)
Large City/Suburb × Income Rank Index						-0.096
						(-0.258,0.066)
Constant	4.469***	6.841***	6.950***	6.994***	7.162***	6.985***
	(3.292,5.647)	(5.310,8.372)	(5.427,8.473)	(5.464,8.525)	(5.652,8.673)	(5.474,8.495)
Individual Level Controls	YES	YES	YES	YES	YES	YES
Country Level Controls	YES	YES	YES	YES	YES	YES
Observations	97,339	97,339	97,024	97,339	97,140	97,134
R^2	0.179	0.181	0.181	0.181	0.181	0.181

Note: OLS regression estimates of the interaction effect of income rank and individual demographics. All columns include the full set of individual and country level controls of the baseline model. However, to ease interpretation, only the specific level and interaction terms tested on each column are displayed. Inference is based on two-sided t-tests with standard errors clustered at the country level; 95% confidence intervals are shown in parentheses. No multiple-comparison adjustments applied. *p<0.05; **p<0.01; ***p<0.001.

Supplementary Table 21: Individual Level Moderators of the Income Rank Effect - Confidence and Trust in Society and Institutions

	(1)	(2)	(3)	(4)	(5)	(6)
Income Rank Index (0-1)	1.157***	1.347***	1.104***	1.280***	1.345***	1.392***
	(0.882,1.433)	(1.181,1.513)	(0.909,1.298)	(1.017,1.542)	(1.184,1.505)	(1.162,1.622)
Law and Order Index						
Law and Order Index (0-100)	0.009***					
	(0.007,0.012)					
Law and Order Index (0-100) × Income Rank Index	-0.001					
	(-0.004,0.002)					
National Institutions Index						
National Institutions Index (0-100)		0.009***				
		(0.006,0.011)				
National Institutions Index (0-100) × Income Rank Index		-0.004*				
		(-0.007,-0.001)				
Corruption Index						
Corruption Index (0-100)			-0.003***			
			(-0.005,-0.002)			
Corruption Index (0-100) × Income Rank Index			0.000			
			(-0.001,0.002)			
Social Life Index						
Social Life Index (0-100)				0.015***		
				(0.013,0.017)		
Social Life Index (0-100) × Income Rank Index				-0.004**		
				(-0.006,-0.001)		
Civic Engagement Index						
Civic Engagement Index (0-100)					0.008***	
					(0.006,0.010)	
Civic Engagement Index (0-100) × Income Rank Index					-0.006***	
					(-0.008,-0.004)	
Community Basics Index						
Community Basics Index (0-100)						0.016***
						(0.013,0.019)
Community Basics Index (0-100) × Income Rank Index						-0.005***
						(-0.008,-0.002)
Constant	6.035***	6.879***	6.960***	5.514***	6.828***	5.564***
	(4.535,7.535)	(5.447,8.312)	(5.355,8.566)	(4.019,7.009)	(5.298,8.358)	(4.057,7.072)
Individual Level Controls	YES	YES	YES	YES	YES	YES
Country Level Controls	YES	YES	YES	YES	YES	YES
Observations	97,339	86,732	87,694	97,339	97,339	97,339
R^2	0.188	0.174	0.183	0.205	0.185	0.204

Note: OLS regression estimates of the interaction effect of income rank and individual level indicators. All columns include the full set of individual and country level controls of the baseline model. However, to ease interpretation, only the specific level and interaction terms tested on each column are displayed. Inference is based on two-sided t-tests with standard errors clustered at the country level; 95% confidence intervals are shown in parentheses. No multiple-comparison adjustments applied. *p<0.05; **p<0.01; ***p<0.001.

Supplementary Table 22: Individual Level Moderators of the Income Rank Effect - Social Preferences

	(1)	(2)	(3)	(4)	(5)	(6)
Income Rank Index (0-1)	1.130***	1.121***	1.150***	1.159***	1.133***	1.165***
	(0.980,1.281)	(0.972,1.271)	(0.997,1.304)	(1.007,1.310)	(0.981,1.284)	(1.013,1.317)
Patience						
Patience	0.206***					
	(0.131,0.281)					
Patience × Income Rank Index	-0.041					
	(-0.131,0.050)					
Risk Taking						
Risk Taking		0.289***				
		(0.212,0.366)				
Risk Taking × Income Rank Index		-0.160***				
		(-0.250,-0.071)				
Positive Reciprocity						
Positive Reciprocity			0.028			
			(-0.050,0.106)			
Positive Reciprocity × Income Rank Index			0.075			
			(-0.024,0.174)			
Negative Reciprocity						
Negative Reciprocity				0.094**		
				(0.026,0.163)		
Negative Reciprocity × Income Rank Index				-0.044		
				(-0.130,0.043)		
Altruism						
Altruism					0.121**	
					(0.042,0.200)	
Altruism × Income Rank Index					0.047	
					(-0.036,0.130)	
Trust						
Trust						0.161***
						(0.086,0.236)
Trust × Income Rank Index						-0.000
						(-0.089,0.089)
Constant	5.041***	5.232***	5.104***	5.005***	5.024***	5.105***
	(3.568,6.513)	(3.744,6.720)	(3.603,6.605)	(3.531,6.479)	(3.509,6.540)	(3.591,6.619)
Individual Level Controls	YES	YES	YES	YES	YES	YES
Country Level Controls	YES	YES	YES	YES	YES	YES
Observations	68,365	68,336	68,711	67,374	68,490	67,609
R^2	0.228	0.231	0.223	0.224	0.226	0.227

Note: OLS regression estimates of the interaction effect of income rank and individual level indicators. All columns include the full set of individual and country level controls of the baseline model. However, to ease interpretation, only the specific level and interaction terms tested on each column are displayed. Variables are standardized. Inference is based on two-sided t-tests with standard errors clustered at the country level; 95% confidence intervals are shown in parentheses. No multiple-comparison adjustments applied. *p<0.05; **p<0.01; ***p<0.001.

Supplementary Table 23: Income Effects on Life Evaluation, Pooling Countries

	Life Evaluation				
	(1)	(2)	(3)	(4)	(5)
Log Income $1000	0.663	0.337			0.068
	(0.570,0.756)	(0.244,0.430)			(-0.129,0.266)
	p = 0.000***	p = 0.000***			p = 0.499
Income Rank Index (0-1)			1.385	1.115	0.966
			(1.239,1.531)	(0.969,1.260)	(0.493,1.438)
			p = 0.000***	p = 0.000***	p = 0.000***
Female		-0.070		-0.024	-0.028
		(-1.072,0.932)		(-1.018,0.971)	(-1.026,0.969)
		p = 0.892		p = 0.963	p = 0.956
Education (Base: Elementary)					
College Degree		0.540		0.494	0.489
		(0.387,0.693)		(0.328,0.659)	(0.329,0.649)
		p = 0.000***		p = 0.000***	p = 0.000***
Secondary Education		0.311		0.292	0.289
		(0.166,0.456)		(0.138,0.447)	(0.139,0.440)
		p = 0.000***		p = 0.001***	p = 0.001***
Unreported		0.453		0.443	0.443
		(0.160,0.746)		(0.158,0.728)	(0.156,0.729)
		p = 0.003**		p = 0.003**	p = 0.003**
Employment Status (Base: Employed Full Time)					
Employed Full Time for Self		-0.020		-0.011	-0.010
		(-0.117,0.078)		(-0.109,0.087)	(-0.107,0.088)
		p = 0.694		p = 0.822	p = 0.849
Employed Part Time (do not want full time)		0.136		0.127	0.134
		(0.042,0.230)		(0.025,0.229)	(0.040,0.228)
		p = 0.005**		p = 0.015*	p = 0.006**
Employed Part Time (want full time)		-0.152		-0.172	-0.161
		(-0.254,-0.050)		(-0.285,-0.059)	(-0.263,-0.059)
		p = 0.004**		p = 0.003**	p = 0.002**
Unemployed		-0.593		-0.583	-0.575
		(-0.718,-0.468)		(-0.714,-0.452)	(-0.701,-0.450)
		p = 0.000***		p = 0.000***	p = 0.000***
Out of Workforce		-0.112		-0.084	-0.083
		(-0.205,-0.019)		(-0.182,0.014)	(-0.180,0.014)
		p = 0.019*		p = 0.093	p = 0.092
Health Problems (Base: Yes)					
No		0.557		0.548	0.546
		(0.459,0.656)		(0.453,0.644)	(0.451,0.642)
		p = 0.000***		p = 0.000***	p = 0.000***
Unreported		0.145		0.156	0.157
		(-0.169,0.459)		(-0.157,0.470)	(-0.157,0.471)
		p = 0.366		p = 0.329	p = 0.327
Marital Status (Base: Single)					
Married		0.024		0.043	0.034
		(-0.082,0.130)		(-0.066,0.153)	(-0.071,0.140)
		p = 0.662		p = 0.436	p = 0.522
Separated		-0.168		-0.186	-0.182
		(-0.302,-0.034)		(-0.322,-0.049)	(-0.319,-0.045)
		p = 0.015*		p = 0.008**	p = 0.010**
Divorced		-0.154		-0.112	-0.116
		(-0.275,-0.032)		(-0.232,0.008)	(-0.234,0.002)
		p = 0.014*		p = 0.068	p = 0.055
Widowed		-0.135		-0.095	-0.100
		(-0.260,-0.010)		(-0.221,0.032)	(-0.223,0.022)
		p = 0.034*		p = 0.145	p = 0.109
Domestic Partner		0.289		0.234	0.241
		(0.166,0.413)		(0.114,0.355)	(0.120,0.362)
		p = 0.000***		p = 0.001***	p = 0.000***
Unreported		0.258		0.293	0.290
		(-0.155,0.671)		(-0.113,0.700)	(-0.117,0.697)
		p = 0.221		p = 0.157	p = 0.163
Urban Area (Base: Rural Area)					
Small Town		-0.009		-0.013	-0.015
		(-0.149,0.131)		(-0.156,0.129)	(-0.156,0.127)
		p = 0.897		p = 0.855	p = 0.841
Large City		-0.023		-0.005	-0.013
		(-0.155,0.110)		(-0.141,0.131)	(-0.144,0.118)
		p = 0.737		p = 0.946	p = 0.846
Suburb of a Large City		0.016		0.034	0.026
		(-0.141,0.174)		(-0.126,0.195)	(-0.130,0.182)
		p = 0.840		p = 0.675	p = 0.746
Unreported		-0.482		-0.439	-0.449
		(-0.820,-0.144)		(-0.772,-0.105)	(-0.784,-0.113)
		p = 0.006**		p = 0.010**	p = 0.009**
Country Level Controls					
Ln Health Expenditure Per Capita $1000		0.066		0.295	0.250
		(-0.145,0.277)		(0.095,0.496)	(-0.013,0.514)
		p = 0.540		p = 0.004**	p = 0.063
Unemployment (% of total labor force)		-0.038		-0.046	-0.044
		(-0.072,-0.004)		(-0.079,-0.013)	(-0.078,-0.011)
		p = 0.028*		p = 0.007**	p = 0.010**
Urban Population (% of total population)		0.016		0.017	0.017
		(0.005,0.026)		(0.006,0.028)	(0.006,0.027)
		p = 0.004**		p = 0.003**	p = 0.003**
Gini Index (0-100)		0.002		-0.012	-0.009
		(-0.023,0.026)		(-0.036,0.013)	(-0.034,0.016)
		p = 0.895		p = 0.342	p = 0.470
Absolute Redistribution		0.003		0.000	0.001
		(-0.021,0.027)		(-0.023,0.023)	(-0.022,0.024)
		p = 0.802		p = 0.981	p = 0.948
Constant	4.216	6.540	5.053	6.998	6.840
	(3.903,4.529)	(4.991,8.089)	(4.812,5.295)	(5.478,8.518)	(5.248,8.433)
	p = 0.000***	p = 0.000***	p = 0.000***	p = 0.000***	p = 0.000***
Observations	97,339	97,339	97,339	97,339	97,339
R^2	0.121	0.177	0.026	0.181	0.181

Note: OLS models for the effect of income and income rank on life evaluation (for survey years 2023-2024). Columns 2, 4, and 5 incorporate individual-level controls, including age, gender (a four-degree polynomial of age and its interaction with gender), and country-level controls. Outliers below the 5 percentile and above the 95 percentile of incomes are excluded. Inference is based on two-sided t-tests with standard errors clustered at the country level; 95% confidence intervals are shown in parentheses. No multiple-comparison adjustments applied. $^{*}p<0.05$; $^{**}p<0.01$; $^{***}p<0.001$.

Supplementary Table 24: Income Effects on Life Evaluation in 5 Years, Pooling Countries

	Life Evaluation in 5 Years				
	(1)	(2)	(3)	(4)	(5)
Log Income $1000	0.288	0.338			0.122
	(0.183,0.394)	(0.234,0.441)			(-0.122,0.366)
	p = 0.000***	p = 0.000***			p = 0.327
Income Rank Index (0-1)			1.564	1.042	0.778
			(1.388,1.741)	(0.862,1.222)	(0.166,1.390)
			p = 0.000***	p = 0.000***	p = 0.013*
Female		-0.059		-0.011	-0.021
		(-1.226,1.108)		(-1.168,1.146)	(-1.181,1.140)
		p = 0.921		p = 0.985	p = 0.973
Education (Base: Elementary)					
College Degree		0.668		0.634	0.625
		(0.478,0.857)		(0.425,0.842)	(0.425,0.825)
		p = 0.000***		p = 0.000***	p = 0.000***
Secondary Education		0.431		0.418	0.413
		(0.243,0.620)		(0.215,0.621)	(0.217,0.610)
		p = 0.000***		p = 0.000***	p = 0.000***
Unreported		0.413		0.409	0.408
		(-0.075,0.902)		(-0.084,0.903)	(-0.084,0.900)
		p = 0.098		p = 0.104	p = 0.105
Employment Status (Base: Employed Full Time)					
Employed Full Time for Self		0.123		0.128	0.131
		(0.023,0.223)		(0.027,0.229)	(0.032,0.231)
		p = 0.016*		p = 0.013*	p = 0.010**
Employed Part Time (do not want full time)		0.103		0.089	0.101
		(-0.002,0.209)		(-0.027,0.205)	(-0.006,0.207)
		p = 0.055		p = 0.133	p = 0.064
Employed Part Time (want full time)		0.078		0.050	0.070
		(-0.035,0.191)		(-0.073,0.173)	(-0.040,0.181)
		p = 0.176		p = 0.426	p = 0.213
Unemployed		-0.318		-0.317	-0.304
		(-0.502,-0.133)		(-0.518,-0.116)	(-0.493,-0.114)
		p = 0.001***		p = 0.003**	p = 0.002**
Out of Workforce		-0.163		-0.142	-0.141
		(-0.285,-0.042)		(-0.272,-0.012)	(-0.269,-0.013)
		p = 0.009**		p = 0.033*	p = 0.031*
Health Problems (Base: Yes)					
No		0.559		0.553	0.549
		(0.461,0.658)		(0.462,0.644)	(0.456,0.643)
		p = 0.000***		p = 0.000***	p = 0.000***
Unreported		0.018		0.019	0.023
		(-0.402,0.439)		(-0.397,0.435)	(-0.394,0.440)
		p = 0.933		p = 0.929	p = 0.914
Marital Status (Base: Single)					
Married		-0.122		-0.099	-0.115
		(-0.237,-0.007)		(-0.215,0.017)	(-0.227,-0.002)
		p = 0.038*		p = 0.095	p = 0.046*
Separated		-0.087		-0.109	-0.101
		(-0.220,0.045)		(-0.243,0.025)	(-0.235,0.033)
		p = 0.197		p = 0.113	p = 0.141
Divorced		-0.173		-0.135	-0.143
		(-0.308,-0.038)		(-0.267,-0.003)	(-0.273,-0.013)
		p = 0.013*		p = 0.045*	p = 0.032*
Widowed		-0.189		-0.153	-0.162
		(-0.319,-0.059)		(-0.281,-0.025)	(-0.290,-0.035)
		p = 0.005**		p = 0.020*	p = 0.013*
Domestic Partner		0.228		0.178	0.190
		(0.096,0.360)		(0.047,0.308)	(0.059,0.321)
		p = 0.001***		p = 0.008**	p = 0.005**
Unreported		0.140		0.163	0.159
		(-0.268,0.547)		(-0.238,0.563)	(-0.244,0.561)
		p = 0.502		p = 0.426	p = 0.441
Urban Area (Base: Rural Area)					
Small Town		-0.070		-0.073	-0.075
		(-0.252,0.112)		(-0.262,0.117)	(-0.261,0.112)
		p = 0.449		p = 0.453	p = 0.433
Large City		-0.029		-0.006	-0.021
		(-0.192,0.134)		(-0.182,0.171)	(-0.186,0.145)
		p = 0.728		p = 0.950	p = 0.806
Suburb of a Large City		-0.126		-0.104	-0.119
		(-0.333,0.082)		(-0.325,0.117)	(-0.326,0.089)
		p = 0.236		p = 0.358	p = 0.263
Unreported		-0.331		-0.288	-0.306
		(-0.628,-0.033)		(-0.593,0.016)	(-0.605,-0.006)
		p = 0.030*		p = 0.064	p = 0.046*
Country Level Controls					
Ln Health Expenditure Per Capita $1000		-0.309		-0.079	-0.160
		(-0.570,-0.048)		(-0.324,0.165)	(-0.503,0.183)
		p = 0.021*		p = 0.526	p = 0.360
Unemployment (% of total labor force)		-0.030		-0.038	-0.035
		(-0.067,0.007)		(-0.073,-0.002)	(-0.073,0.003)
		p = 0.117		p = 0.038*	p = 0.069
Urban Population (% of total population)		0.023		0.024	0.024
		(0.011,0.036)		(0.011,0.037)	(0.011,0.036)
		p = 0.001***		p = 0.001***	p = 0.001***
Gini Index (0-100)		0.023		0.010	0.015
		(-0.003,0.049)		(-0.017,0.037)	(-0.013,0.042)
		p = 0.081		p = 0.465	p = 0.298
Absolute Redistribution		0.003		0.000	0.001
		(-0.023,0.029)		(-0.026,0.026)	(-0.025,0.027)
		p = 0.807		p = 0.996	p = 0.935
Constant	6.350	5.669	6.244	6.181	5.900
	(6.004,6.695)	(3.884,7.455)	(6.022,6.465)	(4.441,7.920)	(3.993,7.808)
	p = 0.000***	p = 0.000***	p = 0.000***	p = 0.000***	p = 0.000***
Observations	90,952	90,952	90,952	90,952	90,952
R^2	0.023	0.135	0.032	0.137	0.137

Note: OLS models for the effect of income and income rank on life evaluation in five years (for survey years 2023-2024). Columns 2, 4, and 5 incorporate individual-level controls, including age, gender (a four-degree polynomial of age and its interaction with gender), and country-level controls. Outliers below the 5 percentile and above the 95 percentile of incomes are excluded. Inference is based on two-sided t-tests with standard errors clustered at the country level; 95% confidence intervals are shown in parentheses. No multiple-comparison adjustments applied. $^{*}p<0.05$; $^{**}p<0.01$; $^{***}p<0.001$.

Supplementary Table 25: Income Effects on Positive Affect, Pooling Countries

	Positive Affect				
	(1)	(2)	(3)	(4)	(5)
Log Income $1000	0.019<br>(0.006,0.031)<br>p = 0.005**	0.020<br>(0.001,0.039)<br>p = 0.042*			-0.025<br>(-0.071,0.022)<br>p = 0.296
Income Rank Index (0-1)			0.143<br>(0.127,0.160)<br>p = 0.000***	0.107<br>(0.091,0.123)<br>p = 0.000***	0.161<br>(0.055,0.267)<br>p = 0.003**
Female		0.076<br>(-0.096,0.247)<br>p = 0.387		0.080<br>(-0.090,0.251)<br>p = 0.357	0.082<br>(-0.089,0.253)<br>p = 0.347
Education (Base: Elementary)					
College Degree		0.039<br>(0.013,0.065)<br>p = 0.004**		0.029<br>(0.003,0.054)<br>p = 0.028*	0.031<br>(0.005,0.056)<br>p = 0.021*
Secondary Education		0.021<br>(-0.003,0.044)<br>p = 0.084		0.016<br>(-0.008,0.040)<br>p = 0.191	0.017<br>(-0.007,0.041)<br>p = 0.164
Unreported		0.045<br>(-0.009,0.098)<br>p = 0.104		0.043<br>(-0.011,0.096)<br>p = 0.116	0.043<br>(-0.011,0.096)<br>p = 0.116
Employment Status (Base: Employed Full Time)					
Employed Full Time for Self		0.023<br>(0.009,0.037)<br>p = 0.002**		0.026<br>(0.011,0.040)<br>p = 0.001***	0.025<br>(0.010,0.040)<br>p = 0.001***
Employed Part Time (do not want full time)		0.035<br>(0.019,0.050)<br>p = 0.000***		0.036<br>(0.020,0.053)<br>p = 0.000***	0.034<br>(0.018,0.050)<br>p = 0.000***
Employed Part Time (want full time)		0.022<br>(0.004,0.040)<br>p = 0.017*		0.024<br>(0.003,0.046)<br>p = 0.027*	0.020<br>(0.002,0.038)<br>p = 0.028*
Unemployed		-0.057<br>(-0.079,-0.035)<br>p = 0.000***		-0.052<br>(-0.077,-0.027)<br>p = 0.000***	-0.054<br>(-0.077,-0.032)<br>p = 0.000***
Out of Workforce		-0.024<br>(-0.040,-0.008)<br>p = 0.004**		-0.019<br>(-0.036,-0.003)<br>p = 0.024*	-0.019<br>(-0.036,-0.003)<br>p = 0.023*
Health Problems (Base: Yes)					
No		0.090<br>(0.076,0.104)<br>p = 0.000***		0.087<br>(0.074,0.101)<br>p = 0.000***	0.088<br>(0.075,0.102)<br>p = 0.000***
Unreported		0.009<br>(-0.065,0.082)<br>p = 0.815		0.011<br>(-0.063,0.084)<br>p = 0.776	0.010<br>(-0.063,0.084)<br>p = 0.783
Marital Status (Base: Single)					
Married		-0.011<br>(-0.027,0.006)<br>p = 0.195		-0.012<br>(-0.030,0.005)<br>p = 0.171	-0.009<br>(-0.025,0.006)<br>p = 0.251
Separated		-0.020<br>(-0.041,0.000)<br>p = 0.053		-0.021<br>(-0.042,-0.001)<br>p = 0.041*	-0.023<br>(-0.043,-0.002)<br>p = 0.028*
Divorced		-0.039<br>(-0.057,-0.020)<br>p = 0.000***		-0.034<br>(-0.052,-0.016)<br>p = 0.001***	-0.032<br>(-0.051,-0.014)<br>p = 0.001***
Widowed		-0.051<br>(-0.073,-0.030)<br>p = 0.000***		-0.048<br>(-0.068,-0.027)<br>p = 0.000***	-0.046<br>(-0.066,-0.026)<br>p = 0.000***
Domestic Partner		0.028<br>(0.009,0.047)<br>p = 0.005**		0.023<br>(0.004,0.041)<br>p = 0.020*	0.020<br>(0.002,0.038)<br>p = 0.029*
Unreported		0.025<br>(-0.018,0.068)<br>p = 0.260		0.028<br>(-0.014,0.070)<br>p = 0.189	0.029<br>(-0.012,0.071)<br>p = 0.166
Urban Area (Base: Rural Area)					
Small Town		-0.009<br>(-0.031,0.012)<br>p = 0.399		-0.011<br>(-0.033,0.012)<br>p = 0.351	-0.010<br>(-0.033,0.013)<br>p = 0.378
Large City		-0.035<br>(-0.056,-0.014)<br>p = 0.001***		-0.037<br>(-0.059,-0.014)<br>p = 0.002**	-0.034<br>(-0.054,-0.013)<br>p = 0.002**
Suburb of a Large City		-0.012<br>(-0.034,0.010)<br>p = 0.302		-0.013<br>(-0.036,0.010)<br>p = 0.265	-0.010<br>(-0.033,0.013)<br>p = 0.393
Unreported		-0.094<br>(-0.135,-0.054)<br>p = 0.000***		-0.092<br>(-0.133,-0.051)<br>p = 0.000***	-0.089<br>(-0.130,-0.047)<br>p = 0.000***
Country Level Controls					
Ln Health Expenditure Per Capita $1000		0.003<br>(-0.029,0.034)<br>p = 0.862		0.017<br>(-0.009,0.044)<br>p = 0.208	0.033<br>(-0.014,0.080)<br>p = 0.166
Unemployment (% of total labor force)		-0.004<br>(-0.010,0.002)<br>p = 0.191		-0.005<br>(-0.011,0.001)<br>p = 0.126	-0.005<br>(-0.011,0.001)<br>p = 0.091
Urban Population (% of total population)		0.001<br>(-0.001,0.002)<br>p = 0.360		0.001<br>(-0.001,0.002)<br>p = 0.304	0.001<br>(-0.001,0.002)<br>p = 0.245
Gini Index (0-100)		0.005<br>(0.002,0.008)<br>p = 0.003**		0.004<br>(0.001,0.007)<br>p = 0.009**	0.003<br>(-0.001,0.006)<br>p = 0.099
Absolute Redistribution		-0.002<br>(-0.006,0.001)<br>p = 0.208		-0.002<br>(-0.006,0.001)<br>p = 0.168	-0.003<br>(-0.006,0.001)<br>p = 0.136
Constant	0.668<br>(0.628,0.708)<br>p = 0.000***	0.659<br>(0.436,0.881)<br>p = 0.000***	0.639<br>(0.614,0.664)<br>p = 0.000***	0.652<br>(0.454,0.849)<br>p = 0.000***	0.708<br>(0.472,0.945)<br>p = 0.000***
Observations	96,439	96,439	96,439	96,439	96,439
R^2	0.004	0.048	0.011	0.052	0.053

Note: OLS models for the effect of income and income rank on positive affect (for survey years 2023-2024). Columns 2, 4, and 5 incorporate individual-level controls, including age, gender (a four-degree polynomial of age and its interaction with gender), and country-level controls. Outliers below the 5 percentile and above the 95 percentile of incomes are excluded. Inference is based on two-sided *t*-tests with standard errors clustered at the country level; 95% confidence intervals are shown in parentheses. No multiple-comparison adjustments applied. $^{*}p<0.05$; $^{**}p<0.01$; $^{***}p<0.001$.

Supplementary Table 26: Income Effects on Negative Affect, Pooling Countries

	Negative Affect				
	(1)	(2)	(3)	(4)	(5)
Log Income \$1000	-0.038	-0.026			-0.006
	(-0.047,-0.028)	(-0.037,-0.016)			(-0.031,0.019)
	p = 0.000***	p = 0.000***			p = 0.647
Income Rank Index (0-1)			-0.124	-0.086	-0.073
			(-0.140,-0.108)	(-0.099,-0.073)	(-0.133,-0.014)
			p = 0.000***	p = 0.000***	p = 0.015*
Female		0.133		0.129	0.130
		(0.008,0.258)		(0.004,0.254)	(0.005,0.255)
		p = 0.037*		p = 0.043*	p = 0.042*
Education (Base: Elementary)					
College Degree		-0.054		-0.051	-0.050
		(-0.073,-0.035)		(-0.069,-0.033)	(-0.068,-0.032)
		p = 0.000***		p = 0.000***	p = 0.000***
Secondary Education		-0.047		-0.046	-0.046
		(-0.063,-0.032)		(-0.062,-0.030)	(-0.061,-0.030)
		p = 0.000***		p = 0.000***	p = 0.000***
Unreported		-0.021		-0.020	-0.020
		(-0.054,0.012)		(-0.053,0.013)	(-0.053,0.013)
		p = 0.212		p = 0.231	p = 0.231
Employment Status (Base: Employed Full Time)					
Employed Full Time for Self		0.002		0.001	0.001
		(-0.010,0.014)		(-0.011,0.014)	(-0.011,0.014)
		p = 0.753		p = 0.824	p = 0.843
Employed Part Time (do not want full time)		-0.021		-0.020	-0.021
		(-0.032,-0.010)		(-0.031,-0.009)	(-0.031,-0.010)
		p = 0.001***		p = 0.001***	p = 0.001***
Employed Part Time (want full time)		0.045		0.047	0.046
		(0.031,0.059)		(0.033,0.061)	(0.032,0.059)
		p = 0.000***		p = 0.000***	p = 0.000***
Unemployed		0.081		0.080	0.079
		(0.062,0.099)		(0.060,0.099)	(0.061,0.098)
		p = 0.000***		p = 0.000***	p = 0.000***
Out of Workforce		-0.010		-0.012	-0.012
		(-0.021,0.000)		(-0.023,-0.001)	(-0.023,-0.002)
		p = 0.057		p = 0.028*	p = 0.026*
Health Problems (Base: Yes)					
No		-0.148		-0.148	-0.147
		(-0.160,-0.137)		(-0.160,-0.136)	(-0.159,-0.136)
		p = 0.000***		p = 0.000***	p = 0.000***
Unreported		-0.101		-0.101	-0.102
		(-0.145,-0.057)		(-0.146,-0.057)	(-0.146,-0.057)
		p = 0.000***		p = 0.000***	p = 0.000***
Marital Status (Base: Single)					
Married		0.005		0.003	0.004
		(-0.008,0.018)		(-0.010,0.017)	(-0.008,0.017)
		p = 0.449		p = 0.616	p = 0.511
Separated		0.027		0.029	0.028
		(0.011,0.044)		(0.013,0.045)	(0.012,0.044)
		p = 0.002**		p = 0.001***	p = 0.001***
Divorced		0.014		0.011	0.012
		(-0.004,0.033)		(-0.007,0.029)	(-0.006,0.030)
		p = 0.125		p = 0.220	p = 0.205
Widowed		0.026		0.023	0.024
		(0.010,0.043)		(0.007,0.040)	(0.008,0.040)
		p = 0.002**		p = 0.006**	p = 0.004**
Domestic Partner		0.007		0.011	0.010
		(-0.006,0.020)		(-0.002,0.024)	(-0.002,0.023)
		p = 0.311		p = 0.106	p = 0.112
Unreported		-0.002		-0.005	-0.004
		(-0.051,0.046)		(-0.053,0.044)	(-0.053,0.044)
		p = 0.932		p = 0.848	p = 0.857
Urban Area (Base: Rural Area)					
Small Town		0.012		0.013	0.013
		(-0.003,0.028)		(-0.003,0.029)	(-0.003,0.029)
		p = 0.122		p = 0.119	p = 0.115
Large City		0.026		0.025	0.026
		(0.011,0.042)		(0.009,0.041)	(0.010,0.041)
		p = 0.001***		p = 0.003**	p = 0.002**
Suburb of a Large City		0.035		0.033	0.034
		(0.018,0.052)		(0.016,0.051)	(0.017,0.052)
		p = 0.000***		p = 0.001***	p = 0.001***
Unreported		0.078		0.075	0.076
		(0.014,0.143)		(0.012,0.138)	(0.012,0.139)
		p = 0.017*		p = 0.020*	p = 0.020*
Country Level Controls					
Ln Health Expenditure Per Capita \$1000		0.003		-0.015	-0.011
		(-0.019,0.025)		(-0.036,0.006)	(-0.040,0.018)
		p = 0.784		p = 0.170	p = 0.461
Unemployment (% of total labor force)		0.000		0.001	0.001
		(-0.003,0.004)		(-0.003,0.005)	(-0.003,0.005)
		p = 0.832		p = 0.575	p = 0.625
Urban Population (% of total population)		0.001		0.001	0.001
		(-0.000,0.002)		(-0.000,0.002)	(-0.000,0.002)
		p = 0.123		p = 0.182	p = 0.162
Gini Index (0-100)		0.001		0.002	0.002
		(-0.002,0.004)		(-0.001,0.005)	(-0.001,0.005)
		p = 0.473		p = 0.159	p = 0.221
Absolute Redistribution		-0.000		0.000	0.000
		(-0.003,0.003)		(-0.003,0.003)	(-0.003,0.003)
		p = 0.944		p = 0.951	p = 0.972
Constant	0.398	-0.009	0.374	-0.045	-0.031
	(0.373,0.424)	(-0.192,0.175)	(0.353,0.394)	(-0.232,0.142)	(-0.223,0.160)
	p = 0.000***	p = 0.927	p = 0.000***	p = 0.639	p = 0.748
Observations	96,953	96,953	96,953	96,953	96,953
R^2	0.022	0.085	0.012	0.086	0.086

Note: OLS models for the effect of income and income rank on positive affect (for survey years 2023-2024). Columns 2, 4, and 5 incorporate individual-level controls, including age, gender (a four-degree polynomial of age and its interaction with gender), and country-level controls. Outliers below the 5 percentile and above the 95 percentile of incomes are excluded. Inference is based on two-sided t-tests with standard errors clustered at the country level; 95% confidence intervals are shown in parentheses. No multiple-comparison adjustments applied. *p<0.05; **p<0.01; ***p<0.001.

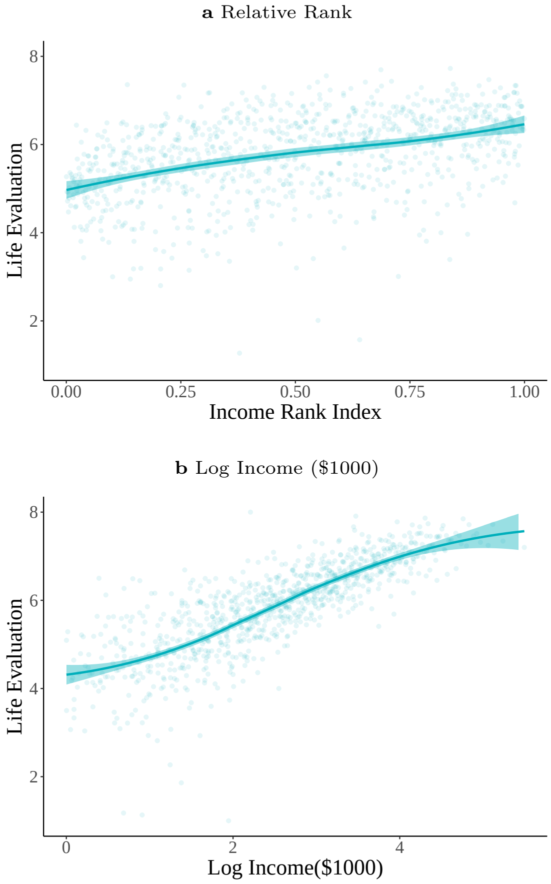

Supplementary Figure 1: **Raw patterns polling data.** **a** Binned scatterplot showing the unconditional relationship between relative income rank (x-axis) and life evaluation (y-axis). **b**, Binned scatterplot showing the corresponding relationship using log income (x-axis). Fitted lines represent local polynomial regressions, and shaded regions denote 95% confidence intervals.

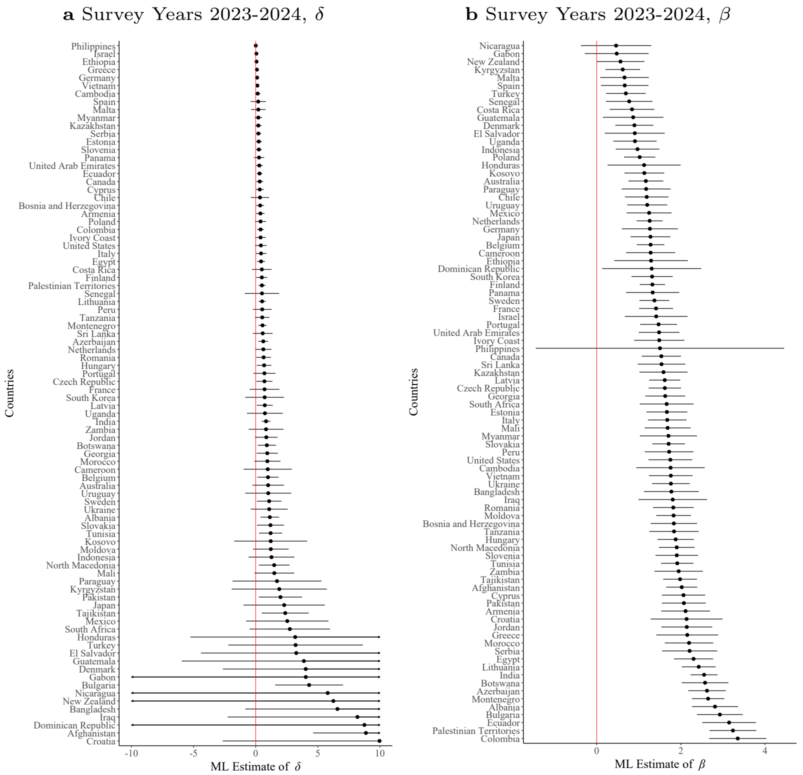

Supplementary Figure 2: **Estimates of δ and β in a generalized-rank model allowing asymmetric comparisons.** **a** Maximum-likelihood estimates of δ in a model that permits asymmetry between upward and downward income comparisons (with $\delta \geq 0$, $\alpha = 1$, and $\gamma = 0$). **b** Corresponding maximum-likelihood estimates of β from the same specification. Estimates outside the boundary of the parameter space are excluded. Lines span 95% CI.

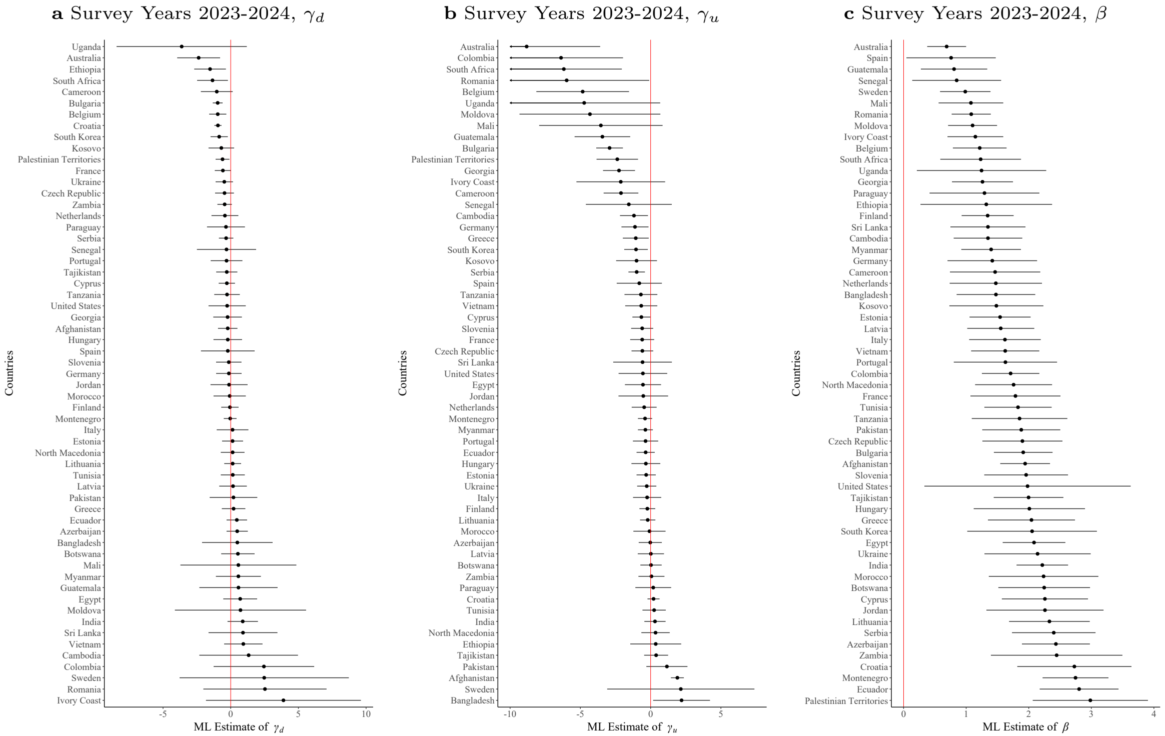

Supplementary Figure 3: **Estimates of γ_d, γ_u, and β in a generalized-rank model with asymmetric distance sensitivity.** **a** Estimates of γ_d, the parameter governing sensitivity to income distances for downward comparisons. **b** Estimates of γ_u, the corresponding parameter for upward comparisons. **c** Estimates of β from the same specification. The model weights upward and downward income comparisons equally ($\delta = 1$, $\alpha = 1$) while allowing separate distance-sensitivity parameters for upward and downward income differences, each restricted to $-10 \leq \gamma_d, \gamma_u \leq 10$. Estimates outside the boundary of the parameter space are excluded. Lines span 95% CI.

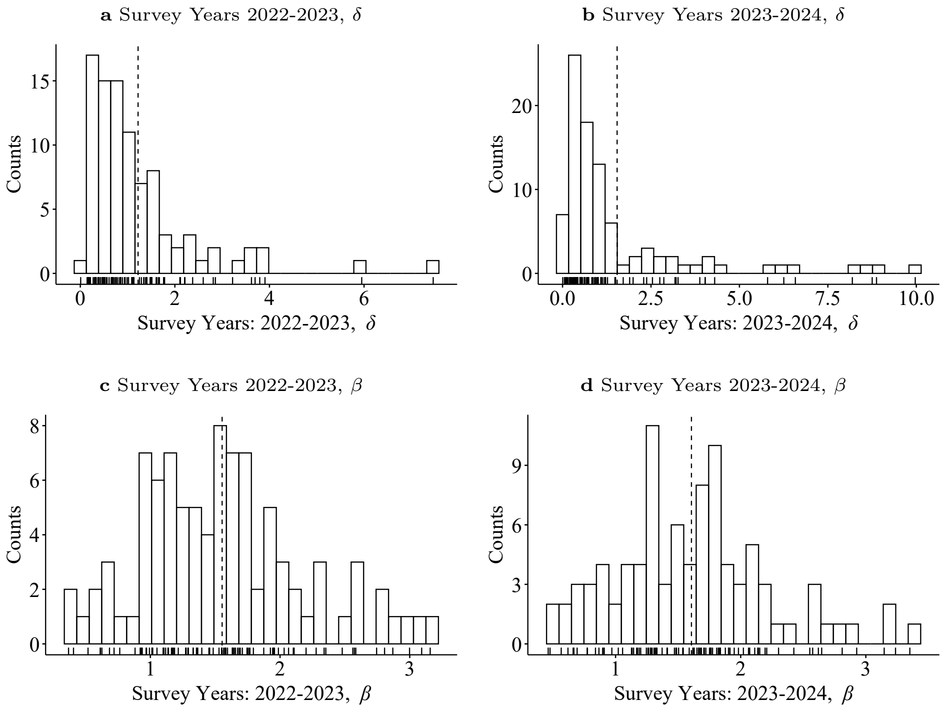

Supplementary Figure 4: **Histograms of δ and β in a generalized-rank model with asymmetric comparison weights.** **a** Histogram of estimates of δ for survey year 2022–2023. **b** Histogram of estimates of δ for survey year 2023–2024. **c** Histogram of estimates of β for survey year 2022–2023. **d** Histogram of estimates of β for survey year 2023–2024. The model allows asymmetry in upward and downward income comparisons by permitting $\delta \geq 0$ while setting $\alpha = 1$ and $\gamma = 0$. Estimates outside the boundary of the parameter space are excluded.

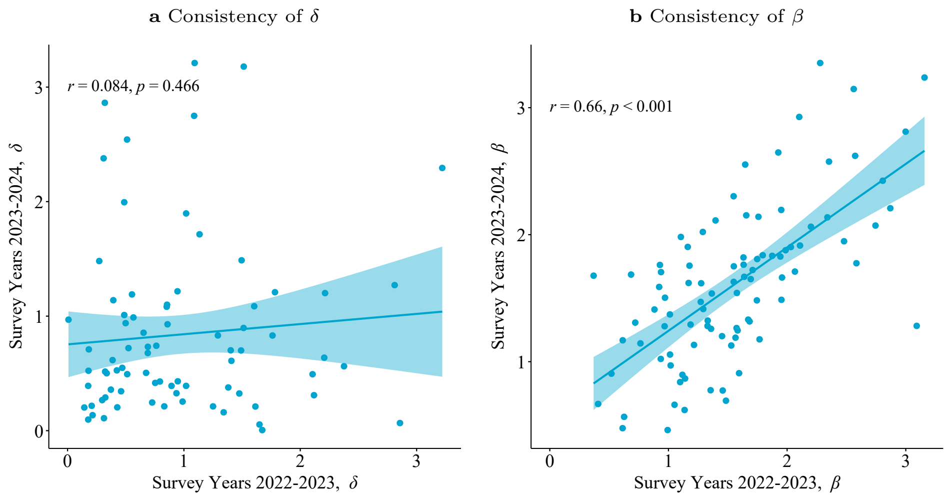

Supplementary Figure 5: **Consistency of estimates across consecutive survey years in a generalized-rank model with asymmetric comparison weights.** **a** Scatter plot of estimates of δ across survey rounds 17 and 18. **b** Scatter plot of estimates of β across the same survey rounds. The model allows asymmetry in upward and downward income comparisons by permitting $\delta \geq 0$ while setting $\alpha = 1$ and $\gamma = 0$. Estimates outside the boundary of the parameter space are excluded. Fitted lines illustrate the best-fit linear relationship between the variables. Shaded areas outline 95% CI. Pearson correlation (r) and associated two-tailed p-values are shown in each panel. Plots include all countries for which the focal parameters could be estimated in both years. n = 92 countries.

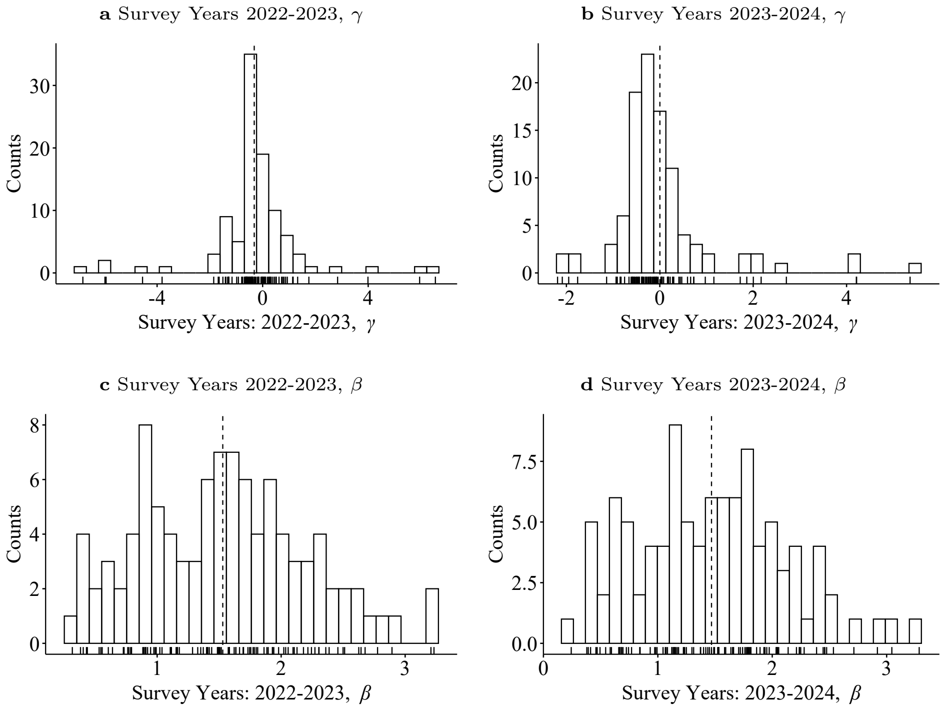

Supplementary Figure 6: **Histograms of γ and β in a generalized-rank model allowing heterogeneous sensitivity to income distance.** **a** Histogram of estimates of γ for survey year 2022–2023. **b** Histogram of estimates of γ for survey year 2023–2024. **c** Histogram of estimates of β for survey year 2022–2023. **d** Histogram of estimates of β for survey year 2023–2024. The model weights upward and downward income comparisons equally ($\delta = 1$, $\alpha = 1$) while allowing the distance-sensitivity parameter γ to vary within $-10 \leq \gamma \leq 10$. Estimates outside the boundary of the parameter space are excluded.

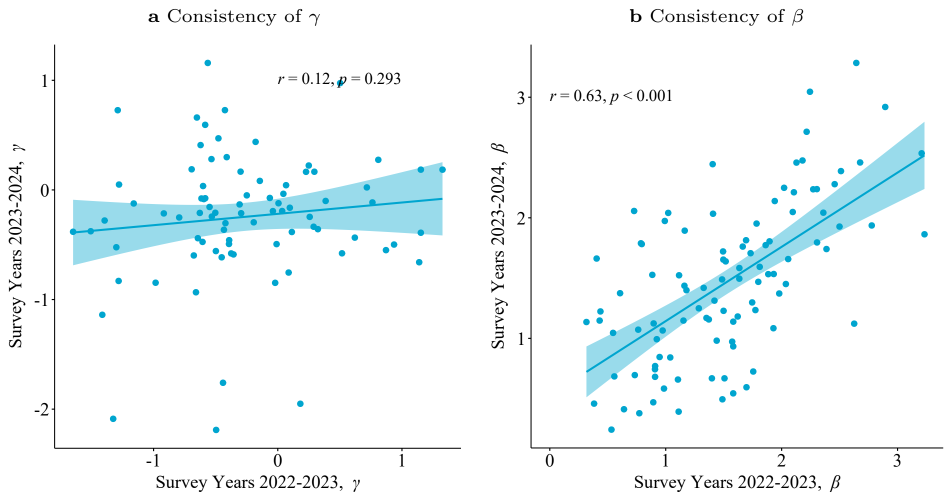

Supplementary Figure 7: Consistency of estimates across consecutive survey years in a generalized-rank model with heterogeneous distance sensitivity. **a** Scatter plot of estimates of γ across survey rounds 17 and 18. **b** Scatter plot of estimates of β across the same survey rounds. The model weights upward and downward income comparisons equally ($\delta = 1$, $\alpha = 1$) while allowing the distance-sensitivity parameter γ to vary within $-10 \leq \gamma \leq 10$. Estimates outside the boundary of the parameter space are excluded. Fitted lines illustrate the best-fit linear relationship between the variables. Shaded areas outline 95% CI. Pearson correlation (r) and associated two-tailed p-values are shown in each panel. Plots include all countries for which the focal parameters could be estimated in both years. n = 100 countries.

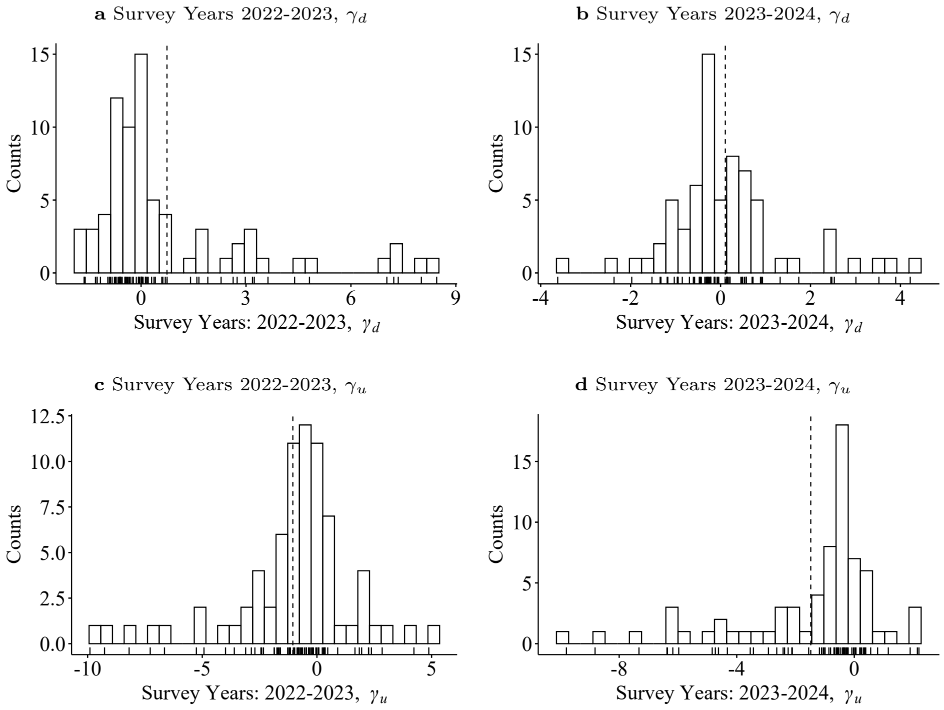

Supplementary Figure 8: **Histograms of γ_d and γ_u in a generalized-rank model with heterogeneous distance sensitivity.** **a** Histogram of estimates of γ_d for survey year 2022–2023. **b** Histogram of estimates of γ_d for survey year 2023–2024. **c** Histogram of estimates of γ_u for survey year 2022–2023. **d** Histogram of estimates of γ_u for survey year 2023–2024. The model weights upward and downward income comparisons equally ($\delta = 1$, $\alpha = 1$) while allowing separate distance-sensitivity parameters for downward and upward comparisons, restricted to $-10 \leq \gamma_d, \gamma_u \leq 10$. Estimates outside the boundary of the parameter space are excluded.

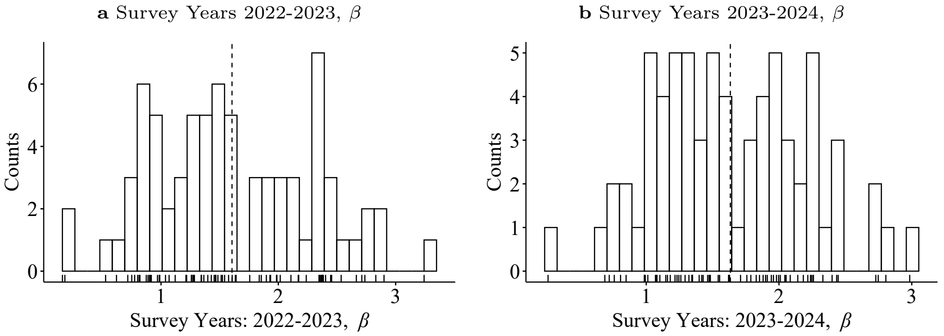

Supplementary Figure 9: **Histograms of β in a generalized-rank model with heterogeneous distance sensitivity. a** Histogram of estimates of β for survey year 2022–2023. **b** Histogram of estimates of β for survey year 2023–2024. The model weights upward and downward income comparisons equally ($\delta = 1$, $\alpha = 1$) while allowing distinct distance-sensitivity parameters for upward and downward comparisons, restricted to $-10 \leq \gamma_d, \gamma_u \leq 10$. Estimates outside the boundary of the parameter space are excluded.

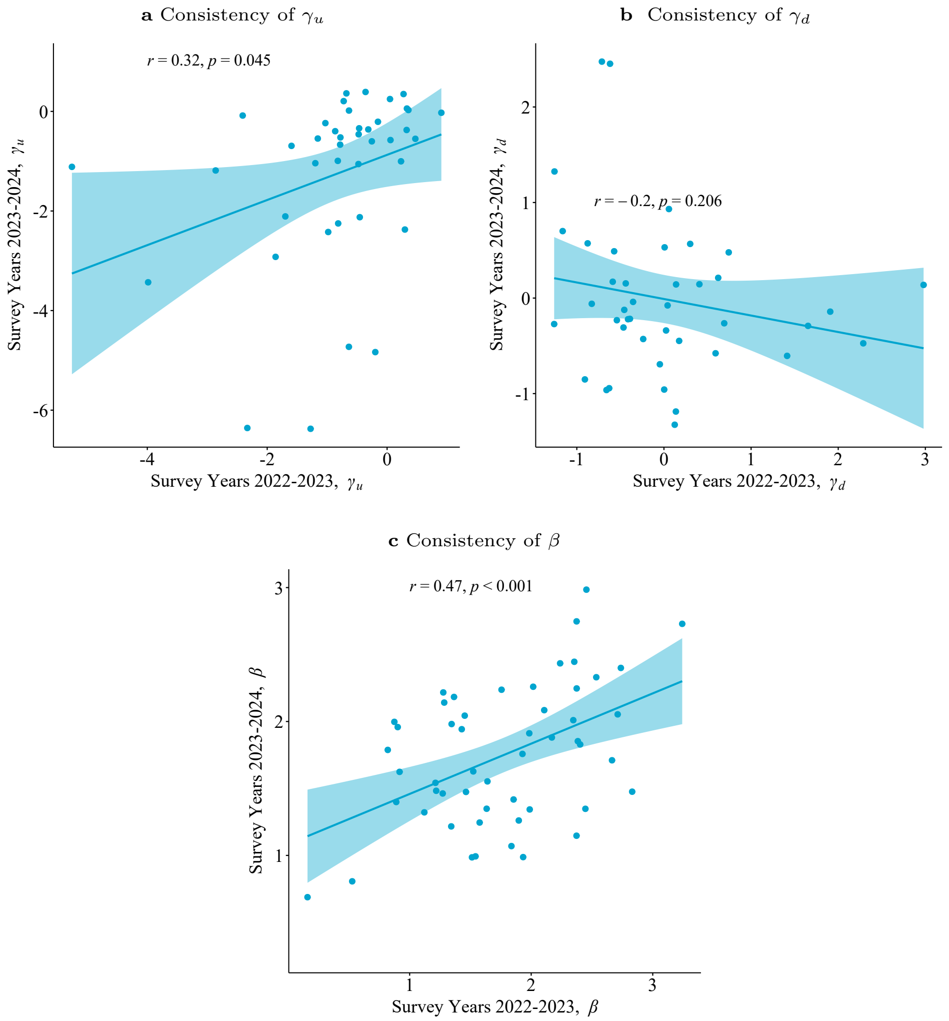

Supplementary Figure 10: **Consistency of estimates across consecutive survey years in a generalized-rank model with heterogeneous distance sensitivity.** **a** Scatter plot of estimates of γ_u across survey rounds 17 and 18. **b** Scatter plot of estimates of γ_d across the same survey rounds. **c** Scatter plot of estimates of β across the same survey rounds. The model weights upward and downward income comparisons equally ($\delta = 1$, $\alpha = 1$) while allowing separate distance-sensitivity parameters for downward and upward comparisons, restricted to $-10 \leq \gamma_d, \gamma_u \leq 10$. Estimates outside the boundary of the parameter space are excluded. Fitted lines illustrate the best-fit linear relationship between the variables. Shaded areas outline 95% CI. Pearson correlation (r) and associated two-tailed p-values are shown in each panel. Plots include all countries for which the focal parameters could be estimated in both years. n = 91 countries.

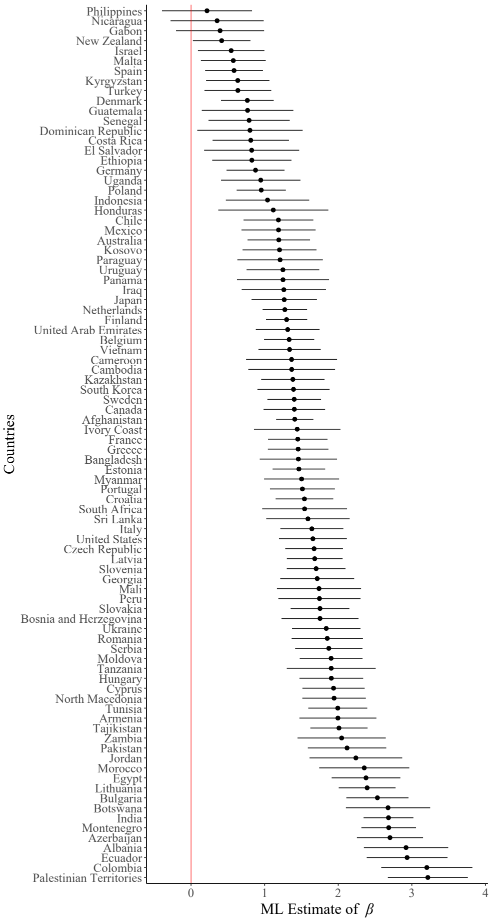

Supplementary Figure 11: **Estimates of β for survey year 2023–2024 in a relative-rank model.** Points represent maximum-likelihood estimates of β for each country, the marginal effect of income rank, obtained from a simplified specification in which upward and downward income comparisons are weighted equally ($\delta = 1$, $\alpha = 1$) and $\gamma = 0$.

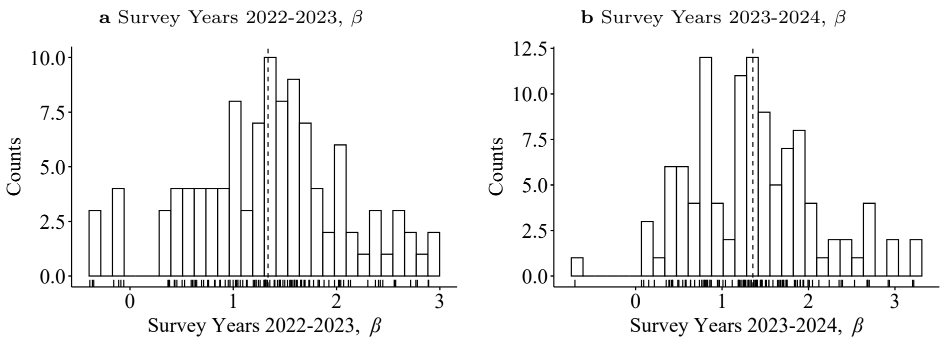

Supplementary Figure 12: **Histograms of β in a relative-rank model. a** Histogram of estimates of β for survey year 2022–2023. **b** Histogram of estimates of β for survey year 2023–2024. Estimates are obtained from a simplified specification in which upward and downward income comparisons are weighted equally ($\delta = 1$, $\alpha = 1$) and $\gamma = 0$.

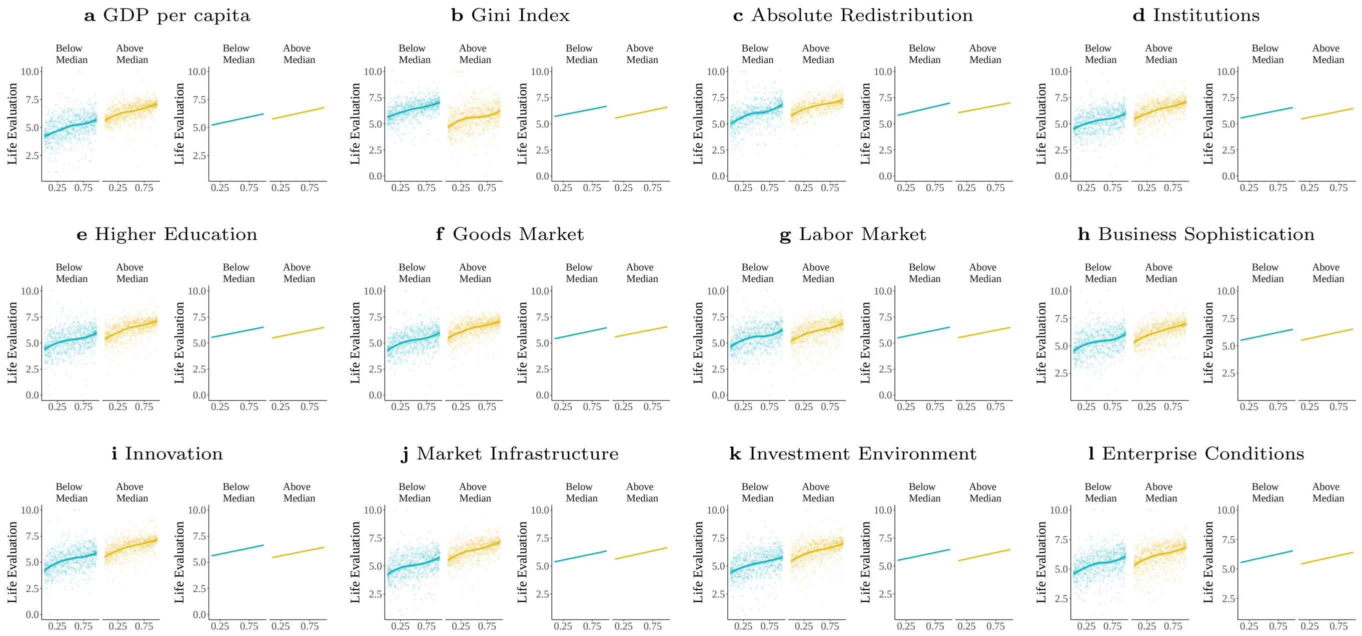

Supplementary Figure 13: **Interaction of Income Rank Coefficient with Country Level Indicators of Economic Development and Subjective Perceptions.** The panels show binned scatter plots of the unconditional relation between the income rank (x-axis) and life evaluation (y-axis) across samples split by country level indicators, followed by fitted patterns of the data (and 95% CI) after the addition of control variables. The fitted patterns on the right side of each panel correspond to percentiles 25 and 75 of the variables under analysis, holding control variables at their mean values or at their mode values for discrete controls.

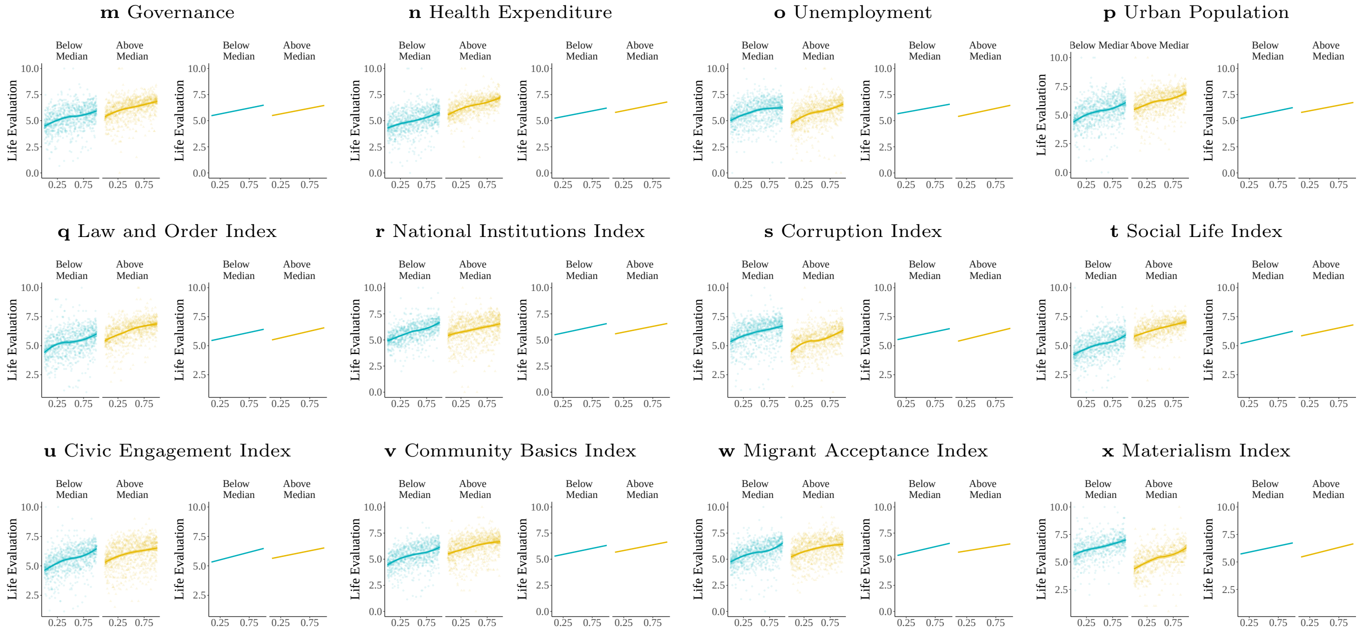

Supplementary Figure 13: **Interaction of Income Rank Coefficient with Country Level Indicators of Economic Development and Subjective Perceptions (Continuation).** The panels show binned scatter plots of the unconditional relation between the income rank (x-axis) and life evaluation (y-axis) across samples split by country level indicators, followed by fitted patterns of the data (and 95% CI) after the addition of control variables. The fitted patterns on the right side of each panel correspond to percentiles 25 and 75 of the variables under analysis, holding control variables at their mean values or at their mode values for discrete controls.

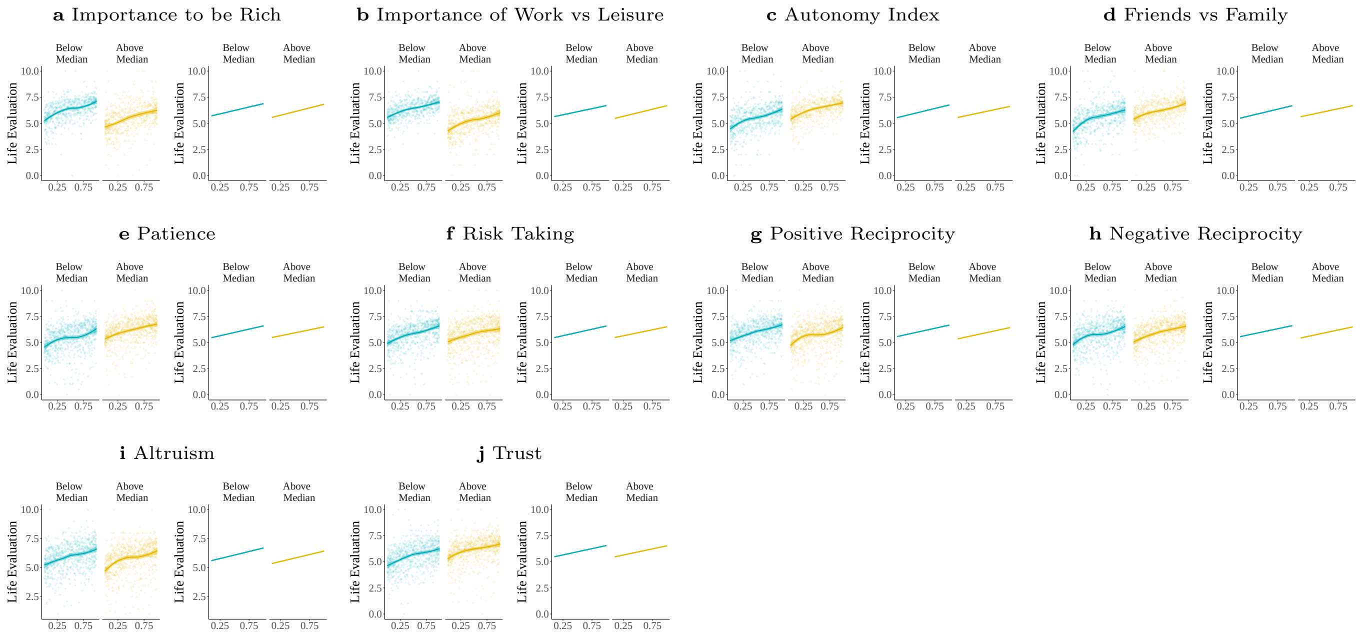

Supplementary Figure 14: **Interaction of Income Rank Coefficient with Country Level Indicators of Individualism and Economic Preferences.** The panels show binned scatter plots of the unconditional relation between the income rank (x-axis) and life evaluation (y-axis) across samples split by country level indicators, followed by fitted patterns of the data (and 95% CI) after the addition of control variables. The fitted patterns on the right side of each panel correspond to percentiles 25 and 75 of the variables under analysis, holding control variables at their mean values or at their mode values for discrete controls.

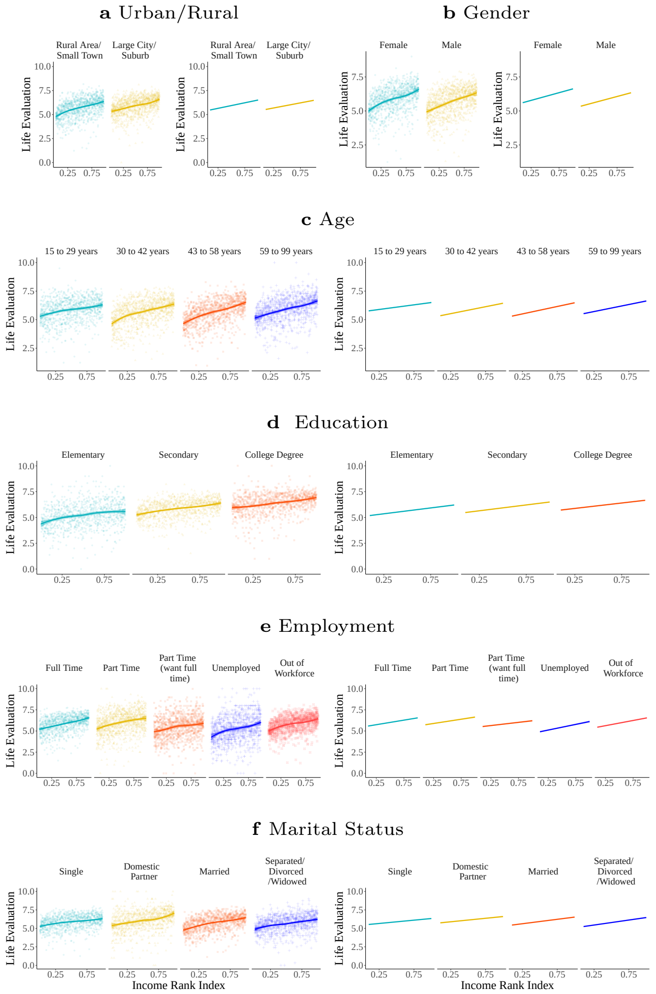

Supplementary Figure 15: **Interaction of Income Rank Coefficient with Demographics: Raw Data and Fitted Data After the Addition of Controls.** The panels show binned scatter plots of the relation between the income rank (x-axis) and life evaluation (y-axis) across samples split by individual demographics, followed by fitted patterns of the data (with 95% CI). Fitted patterns in the right side of each panel are computed for each demographic category, holding control variables at their mean values or at their mode values for discrete controls.

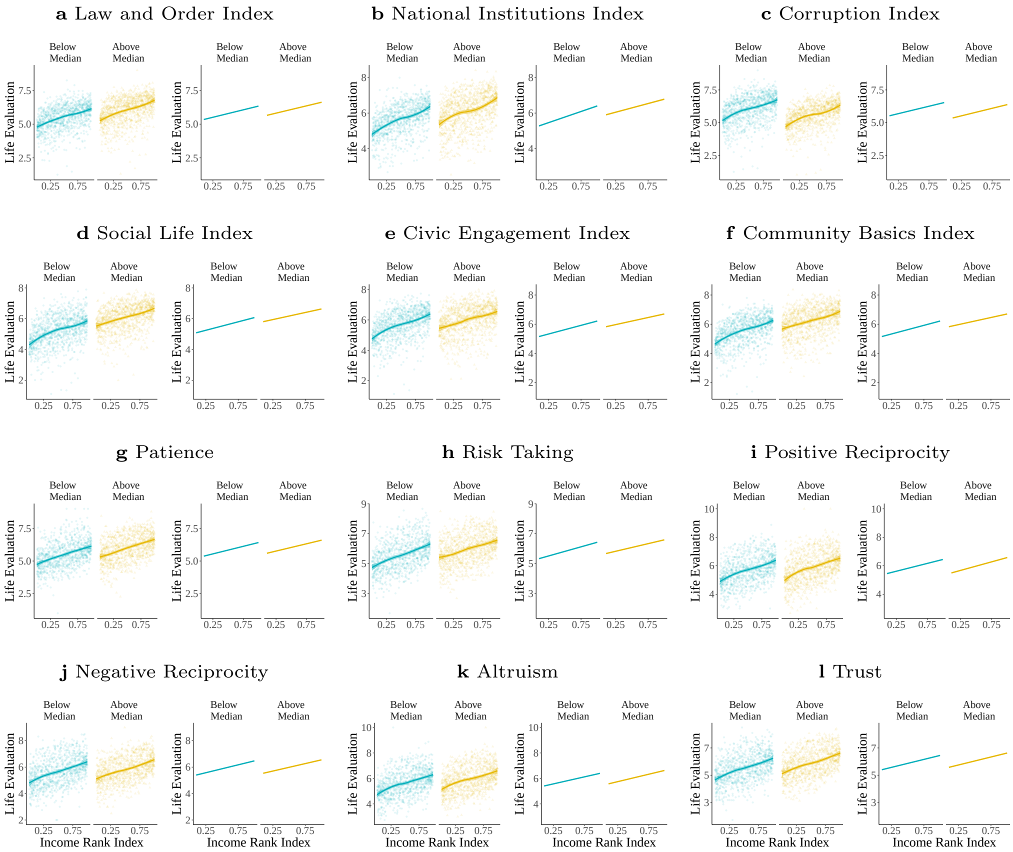

Supplementary Figure 16: **Interaction of Income Rank Coefficient with Individual Level Indicators: Raw Data and Fitted Data After the Addition of Controls.** The panels show binned scatter plots of the relation between the income rank (x-axis) and life evaluation (y-axis) across samples split by individual level indicators, followed by fitted patterns of the data (with 95% CI). Fitted patterns in the right side of each panel correspond to percentiles 25 and 75 of the variables under analysis, holding controls variables at their mean values or at their mode values for discrete controls.

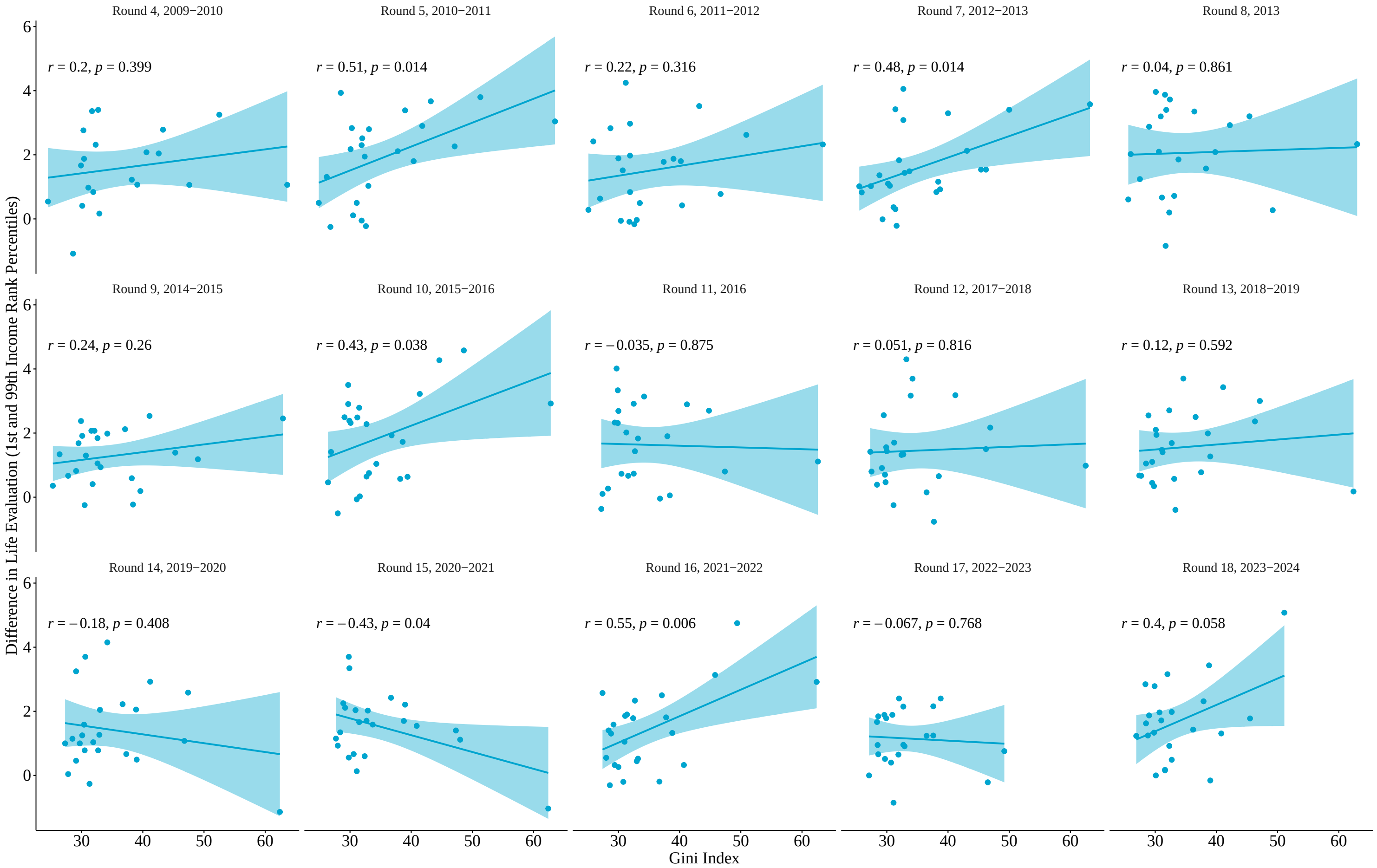

Supplementary Figure 17: **Interaction of Income Rank Coefficient with Gini Index (24 Countries).** The panels show the life evaluation gap between those at the top and bottom 1% in the income rank (y-axis) across different levels of income inequality (x-axis). The plot attempts to replicate the findings of [11] with the sample of 24 countries in the authors' study. For consistency with the authors' methodology, the rank is defined as ratio between the number of people with a lower income than that of the respondent and the total number of respondents in the individual's country of residence $\frac{(i-1)}{(N-1)}$. The patterns show no consistent interaction effects between income rank and income inequality on life evaluation across years. Fitted lines illustrate the best-fit linear relationship between the variables. Pearson correlation (r) and associated two-tailed p-values are shown in each panel. Shaded areas outline 95% CI.

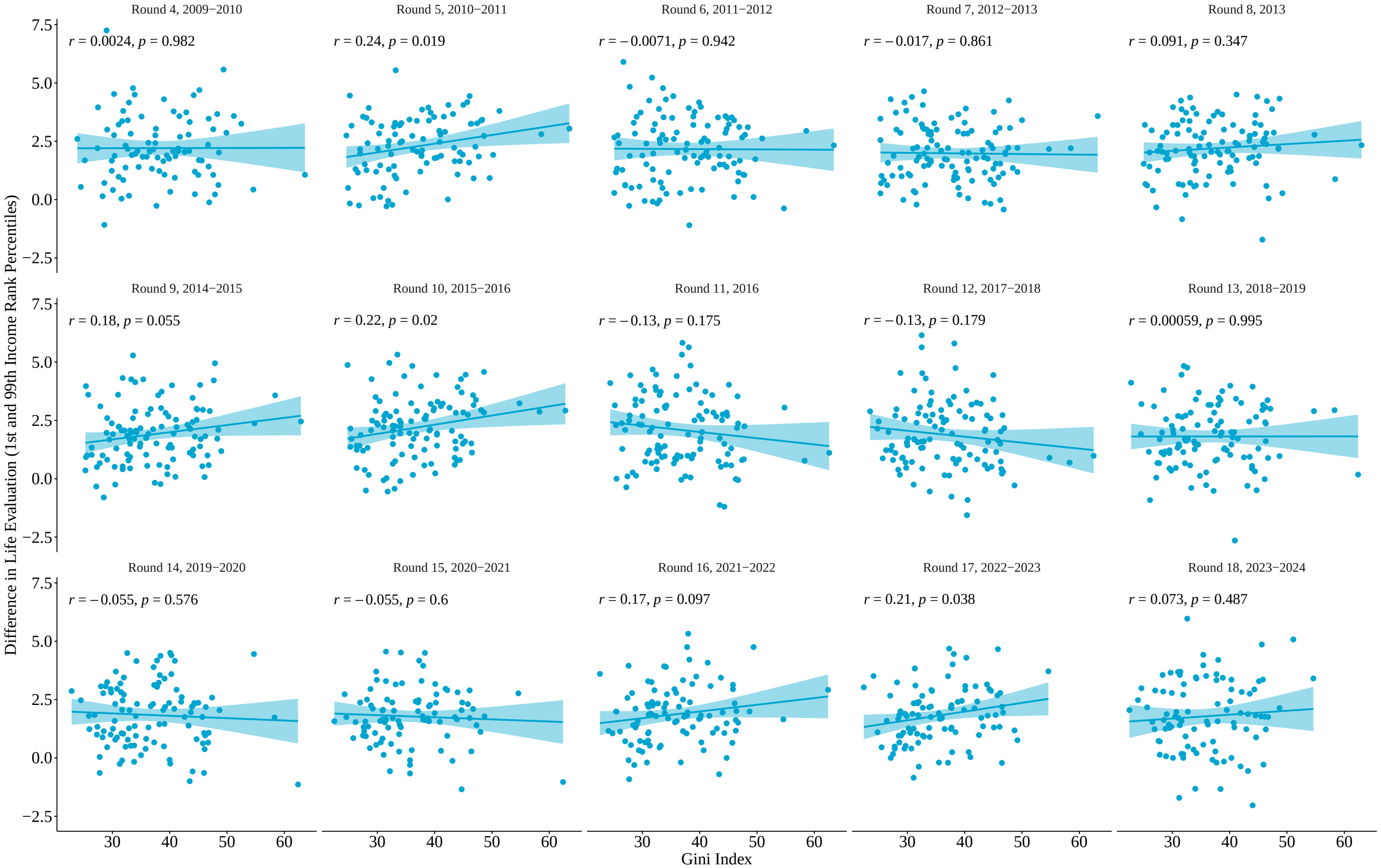

Supplementary Figure 18: **Interaction of Income Rank Coefficient with Gini Index (113 Countries).** The panels show the life evaluation gap between those at the top and bottom 1% in the income rank (y-axis) across different levels of income inequality (x-axis). The plot attempts to replicate the findings of [11] with a larger sample of 113 countries and longer horizon (including the 24 countries in the authors' study). For consistency with the authors' methodology, the rank is defined as ratio between the number of people with a lower income than that of the respondent and the total number of respondents in the individual's country of residence $\frac{(i-1)}{(N-1)}$. The patterns show no consistent interaction effects between income rank and income inequality on life evaluation across years. Fitted lines illustrate the best-fit linear relationship between the variables. Pearson correlation (r) and associated two-tailed p-values are shown in each panel. Shaded areas outline 95% CI.

References

[1] Kifle, T. Do comparison wages play a major role in determining overall job satisfaction? Evidence from Australia. *Journal of Happiness Studies* **15**, 613–638 (2014).

[2] Mujcic, R. & Frijters, P. Economic choices and status: measuring preferences for income rank. *Oxford Economic Papers-New Series* **65**, 47–73 (2013).

[3] Clark, A. E. & Senik, C. *Income comparisons in Chinese villages*, 216–236 (OUP, Oxford, 2014).

[4] Clark, A. E., Kristensen, N. & Westergård-Nielsen, N. Economic satisfaction and income rank in small neighbourhoods. *Journal of the European Economic Association* **7**, 519–527 (2009).

[5] Budria, S. Are relative-income effects constant across the well-being distribution? *Journal of Happiness Studies* **14**, 1379–1408 (2013).

[6] Lakshmanasamy, T. & Maya, K. Rank status and happiness in India: A panel ordered probit estimation of the effect of ordinal relative comparison on well-being. *Indian Journal of Health and Wellbeing* **11** (2020).

[7] Gero, K., Kondo, K., Kondo, N., Shirai, K. & Kawachi, I. Associations of relative deprivation and income rank with depressive symptoms among older adults in Japan. *Social Science & Medicine* **189**, 138–144 (2017).

[8] Acosta-González, H. N. & Marcenaro-Gutiérrez, O. D. What explains life satisfaction? Relative income or rank income? The case of Ecuador. *Applied Economics Letters* 1–5 (2020).

[9] Boyce, C. J., Brown, G. D. A. & Moore, S. C. Money and happiness: Rank of income, not income, affects life satisfaction. *Psychological Science* **21**, 471–475 (2010).

[10] Brown, G. D. A., Gathergood, J. & Weber, J. Relative rank and life satisfaction: Evidence from US households. *SSRN Electronic Journal* (2017).

[11] Macchia, L., Plagnol, A. C. & Powdthavee, N. Buying happiness in an unequal world: Rank of income more strongly predicts well-being in more unequal countries. *Personality and Social Psychology Bulletin* **46**, 769–780 (2020).

[12] Solt, F. Measuring income inequality across countries and over time: The standardized world income inequality database. *Social Science Quarterly* **101**, 1183–1199 (2020).

[13] Inglehart, R. *et al.* World values survey: All rounds – country-pooled datafile. JD Systems Institute & WVSA Secretariat, Madrid, Spain & Vienna, Austria

(2022). Dataset Version 3.0.0.

[14] Santos, H. C., Varnum, M. E. & Grossmann, I. Global increases in individualism. *Psychological Science* **28**, 1228–1239 (2017).

[15] Falk, A. *et al.* Global evidence on economic preferences. *The Quarterly Journal of Economics* **133**, 1645–1692 (2018).

[16] Schwab, K. *et al.* The global competitiveness report 2019 (2019).

[17] Legatum Institute. The 2023 legatum prosperity index (2023).

[18] Tan, J. J. X., Kraus, M. W., Carpenter, N. C. & Adler, N. E. The association between objective and subjective socioeconomic status and subjective well-being: A meta-analytic review. *Psychological Bulletin* **146**, 970–1020 (2020).
