# Supplementary material for: Social status and the relationship between income rank and well-being in 109 nations
Source: Nat Commun. 2026 Feb 20;17:2962. doi: 10.1038/s41467-026-69729-x (PMC13035997; doi:10.1038/s41467-026-69729-x)
Supplement: Supplementary file 2 — Reporting Summary [file 41467_2026_69729_MOESM2_ESM.pdf]

nature portfolio

Corresponding author(s): Edika Quispe-Torreblanca

Last updated by author(s): Nov 28, 2025

Reporting Summary

Nature Portfolio wishes to improve the reproducibility of the work that we publish. This form provides structure for consistency and transparency in reporting. For further information on Nature Portfolio policies, see our Editorial Policies and the Editorial Policy Checklist.

Statistics

For all statistical analyses, confirm that the following items are present in the figure legend, table legend, main text, or Methods section.

n/a	Confirmed	
☐	☒	The exact sample size (*n*) for each experimental group/condition, given as a discrete number and unit of measurement
☒	☐	A statement on whether measurements were taken from distinct samples or whether the same sample was measured repeatedly
☐	☒	The statistical test(s) used AND whether they are one- or two-sided<br>*Only common tests should be described solely by name; describe more complex techniques in the Methods section.*
☐	☒	A description of all covariates tested
☐	☒	A description of any assumptions or corrections, such as tests of normality and adjustment for multiple comparisons
☐	☒	A full description of the statistical parameters including central tendency (e.g. means) or other basic estimates (e.g. regression coefficient) AND variation (e.g. standard deviation) or associated estimates of uncertainty (e.g. confidence intervals)
☐	☒	For null hypothesis testing, the test statistic (e.g. *F*, *t*, *r*) with confidence intervals, effect sizes, degrees of freedom and *P* value noted<br>*Give P values as exact values whenever suitable.*
☒	☐	For Bayesian analysis, information on the choice of priors and Markov chain Monte Carlo settings
☒	☐	For hierarchical and complex designs, identification of the appropriate level for tests and full reporting of outcomes
☒	☐	Estimates of effect sizes (e.g. Cohen's *d*, Pearson's *r*), indicating how they were calculated

Our web collection on statistics for biologists contains articles on many of the points above.

Software and code

Policy information about availability of computer code

Data collection	NA
Data analysis	Our analyses were conducted in R (R version 4.2.0). The study's analysis code is available at https://osf.io/bj9cz

For manuscripts utilizing custom algorithms or software that are central to the research but not yet described in published literature, software must be made available to editors and reviewers. We strongly encourage code deposition in a community repository (e.g. GitHub). See the Nature Portfolio guidelines for submitting code & software for further information.

Data

Policy information about availability of data

All manuscripts must include a data availability statement. This statement should provide the following information, where applicable:

- Accession codes, unique identifiers, or web links for publicly available datasets
- A description of any restrictions on data availability
- For clinical datasets or third party data, please ensure that the statement adheres to our policy

The primary data supporting this study are available from the Gallup Organization (https://www.gallup.com/analytics/318923/world-poll-publicdatasets.aspx) to subscribed researchers or research advisors and may be made temporarily available under controlled conditions for peer review. Additional country-level cultural,

institutional, and economic variables are drawn from publicly available sources: individualism and materialism from the World Values Survey; incentivized measures of economic and social preferences from the Global Preferences Survey; economic competitiveness and openness from the Global Competitiveness Report and the Legatum Institute's Prosperity Index Report; income inequality from the Standardized World Income Inequality Database; and GDP per capita, unemployment, and urban population from the World Bank's World Development Indicators, based on the latest available estimates at the time of download (April 2025), as detailed in Supplementary Note 2.

Research involving human participants, their data, or biological material

Policy information about studies with human participants or human data. See also policy information about sex, gender (identity/presentation), and sexual orientation and race, ethnicity and racism.

Reporting on sex and gender	Sex/gender was not a central focus of the study but was included both as a demographic covariate and, in exploratory moderation analyses, to assess whether the effect of income rank on subjective well-being varied by gender. These analyses were not pre-specified as primary hypotheses but were conducted as part of broader interaction models with multiple individual-level variables. Gender was self-reported in the Gallup World Poll and analysed as a binary variable (male/female). Results are reported in the manuscript (Figure 5) and Supplementary Materials. We did not perform full disaggregated subgroup analyses, as the study was not powered for this and such analyses were not part of the original study design.
Reporting on race, ethnicity, or other socially relevant groupings	Neither race nor ethnicity was used in the study.
Population characteristics	See above.
Recruitment	The study uses secondary data. Data was collected by the Gallup World Poll through structured surveys conducted in multiple countries.
Ethics oversight	This study used anonymized secondary data provided by Gallup and was exempt from ethics approval. Gallup ensures that their data collection processes comply with relevant ethical guidelines and legal requirements in the countries involved.

Note that full information on the approval of the study protocol must also be provided in the manuscript.

Field-specific reporting

Please select the one below that is the best fit for your research. If you are not sure, read the appropriate sections before making your selection.

☐ Life sciences ☒ Behavioural & social sciences ☐ Ecological, evolutionary & environmental sciences

For a reference copy of the document with all sections, see nature.com/documents/nr-reporting-summary-flat.pdf

Behavioural & social sciences study design

All studies must disclose on these points even when the disclosure is negative.

Study description	The study examines whether people's well-being is associated with their relative income deprivation, their social status defined as the ranked position of their income within a community, or their absolute income level. A general model is described that includes various measures of relative income, including relative income deprivation and income rank. The analysis uses quantitative, individual-level secondary data from the Gallup World Poll (GWP), covering six survey rounds conducted between 2013 and 2024. The study design is observational and cross-national, based on statistical analyses of survey responses from 109 countries.
Research sample	The main dataset used in the paper is the Gallup World Poll, an annual, nationally representative survey of adults aged 15 and older. Respondents include both men and women across a wide range of ages, education levels, and socioeconomic backgrounds. For this study, we use data from six survey rounds: Round 8 (2013), Round 9 (2014–2015), Round 12 (2017–2018), Round 13 (2018–2019), Round 17 (2022–2023), and Round 18 (2023–2024). These rounds were selected to create three evenly spaced pairs across the decade, allowing us to assess both short-term (consecutive rounds) and medium-term consistency of income rank effects on well-being.
Sampling strategy	We use the full set of available respondents from the Gallup World Poll, except for the exclusions described in the data exclusions section.
Data collection	This study uses secondary data from the Gallup World Poll. All data were collected by Gallup using standardized survey protocols that include probability-based sampling, structured questionnaires, and face-to-face or telephone interviews depending on country context. The survey instruments record self-reported measures of well-being, household income, and a range of demographic variables. Details on Gallup's data collection procedures, sampling design, and survey instruments are documented at: https://www.gallup.com/178667/gallup-world-poll-work.aspx
Timing	The study uses secondary data from the Gallup World Poll across six distinct survey rounds collected over a ten-year period. Gallup conducts annual or biennial data collection in many countries. The data-collection periods used in the study were: Round 8 (2013), Round 9 (2014–2015), Round 12 (2017–2018), Round 13 (2018–2019), Round 17 (2022–2023), and Round 18 (2023–2024). Gallup does not release exact day-level fieldwork dates, but each round corresponds to a defined survey window. As these are secondary

	data, the research team had no involvement in the timing or administration of data collection.
Data exclusions	The original dataset for the six survey rounds we analyse includes 703,965 records from 109 countries. After removing entries missing well-being (life evaluation) or income data, the number reduced to 695,922 records. Further exclusions were made based on missing age information, bringing the count down to 694,290. Finally, we eliminated outliers in income (outside 5th and 95th percentiles) to avoid regression artifacts, resulting in 608,226 observations for analysis.
Non-participation	This study uses secondary data from the Gallup World Poll. Specific non-participation rates are not available to us. The typical survey includes at least 1,000 individuals, with oversamples in major cities or areas of special interest, and sample sizes of at least 2,000 in large countries like China and Russia.
Randomization	The study uses secondary data, and there is no indication of randomization being applied as part of the research design.

Reporting for specific materials, systems and methods

We require information from authors about some types of materials, experimental systems and methods used in many studies. Here, indicate whether each material, system or method listed is relevant to your study. If you are not sure if a list item applies to your research, read the appropriate section before selecting a response.

Materials & experimental systems

n/a	Involved in the study
☒	☐ Antibodies
☒	☐ Eukaryotic cell lines
☒	☐ Palaeontology and archaeology
☒	☐ Animals and other organisms
☒	☐ Clinical data
☒	☐ Dual use research of concern
☒	☐ Plants

Methods

n/a	Involved in the study
☒	☐ ChIP-seq
☒	☐ Flow cytometry
☒	☐ MRI-based neuroimaging

Plants

Seed stocks	NA
Novel plant genotypes	NA
Authentication	NA
